# Supplementary material for: Prime editing for the investigation of aberrant splicing defect associated with a pathogenic PRPH2 variant
Source: Mol Ther Nucleic Acids. 2025 Oct 13;36(4):102740. doi: 10.1016/j.omtn.2025.102740 (PMC12594906; doi:10.1016/j.omtn.2025.102740)
Supplement: Document S1. Figures S1–S8 and Tables S1–S4 [file mmc1.pdf]

OMTN, Volume *36*

Supplemental information

Prime editing for the investigation of aberrant splicing defect associated with a pathogenic *PRPH2* variant

Bruna Lopes da Costa, Kyle M. Helms, Keith Theodore, Yi-Ting Tsai, Salvatore Marco Caruso, Siyuan Liu, Jose Ronaldo Lima de Carvalho, Nicholas D. Nolan, Saleha Tahir, Christopher D. Makinson, Stephen H. Tsang, and Peter M.J. Quinn

Supplemental Material

Supplemental Tables

Table S1 – Spacer and 3'-extension sequences of the pegRNA and nsgRNA constructs

Plasmid name	Spacer (5'-3')	3' Extension (5'-3')
pU6-pegRNA-FANCF+1A>G	GGAATCCCTTCTGCAGCACC	GGAAAAGCGATCCAGGCGCTGCAGAAGGGA
pU6-pegRNA-FANCF+1A>T	GGAATCCCTTCTGCAGCACC	GGAAAAGCGATCCAGGAGCTGCAGAAGGGA
pU6-pegRNA-FANCF+1A>C	GGAATCCCTTCTGCAGCACC	GGAAAAGCGATCCAGGGGCTGCAGAAGGGA
pU6-pegRNA-FANCF+1insA	GGAATCCCTTCTGCAGCACC	GGAAAAGCGATCCAGGTTGCTGCAGAAGG
pU6-pegRNA-FANCF+1insG	GGAATCCCTTCTGCAGCACC	GGAAAAGCGATCCAGGCTGCTGCAGAAGG
pU6-pegRNA-FANCF+1insT	GGAATCCCTTCTGCAGCACC	GGAAAAGCGATCCAGGATGCTGCAGAAGG
pU6-pegRNA-FANCF+1insC	GGAATCCCTTCTGCAGCACC	GGAAAAGCGATCCAGGGTGCTGCAGAAGG
pU6-pegRNA-FANCF+1delA	GGAATCCCTTCTGCAGCACC	GGAAAAGCGATCCAGGGCTGCAGAAGGGA
pU6-pegRNA-FANCF+2delC	GGAATCCCTTCTGCAGCACC	GGAAAAGCGATCCAGTGCTGCAGAAGGGA
pU6-pegRNA-FANCF+2-3delCC	GGAATCCCTTCTGCAGCACC	GGAAAAGCGATCCATGCTGCAGAAGGGA
pU6-pegRNA-FANCF+4delT	GGAATCCCTTCTGCAGCACC	GGAAAAGCGATCCGGTGCTGCAGAAGGGA
pU6-pegRNA-FANCF+5delG	GGAATCCCTTCTGCAGCACC	GGAAAAGCGATCAGGTGCTGCAGAAGGGA
p7SK-nsgRNA-FANCF	GGGGTCCCAGGTGCTGACGT	NOT APPLICABLE
pU6-pegRNA-HEK3+1T>A	GGCCCAGACTGAGCACGTGA	TCCTCTGCCATCTCGTGCTCAGTCTG
pU6-pegRNA-HEK3+1T>C	GGCCCAGACTGAGCACGTGA	TCCTCTGCCATCGCGTGCTCAGTCTG
pU6-pegRNA-HEK3+1T>G	GGCCCAGACTGAGCACGTGA	TCCTCTGCCATCCCGTGCTCAGTCTG
pU6-pegRNA-HEK3+1insA	GGCCCAGACTGAGCACGTGA	TCCTCTGCCATCATCGTGCTCAGTCTG
pU6-pegRNA-HEK3+1insG	GGCCCAGACTGAGCACGTGA	TCCTCTGCCATCACCGTGCTCAGTCTG
pU6-pegRNA-HEK3+1insT	GGCCCAGACTGAGCACGTGA	TCCTCTGCCATCAACGTGCTCAGTCTG
pU6-pegRNA-HEK3+1insC	GGCCCAGACTGAGCACGTGA	TCCTCTGCCATCAGCGTGCTCAGTCTG
pU6-pegRNA-HEK3+1delT	GGCCCAGACTGAGCACGTGA	TCCTCTGCCATCCGTGCTCAGTCTG
pU6-pegRNA-HEK3+2delG	GGCCCAGACTGAGCACGTGA	TCCTCTGCCATACGTGCTCAGTCTG
pU6-pegRNA-HEK3+3delA	GGCCCAGACTGAGCACGTGA	TCCTCTGCCACACGTGCTCAGTCTG
pU6-pegRNA-HEK3+4delT	GGCCCAGACTGAGCACGTGA	TCCTCTGCCTCACGTGCTCAGTCTG
pU6-pegRNA-HEK3+5delG	GGCCCAGACTGAGCACGTGA	TCCTCTGCATCACGTGCTCAGTCTG
pU6-pegRNA-HEK3+1CTTins	GGCCCAGACTGAGCACGTGA	TCCTCTGCCATCAAAGCGTGCTCAGTCTG
p7SK-nsgRNA-HEK3	GTCAACCAGTATCCCGGTGC	NOT APPLICABLE
pU6-pegRNA-PRPH2-828+1G>A-knock-in	GCTCCTCATTTGGCTCTTCG	GGGCCTATCTCGAAGAGCCAAATGAGG

p7SK-nsgRNA-PRPH2-828+1G>A-knock-in	GCTGCTGTAGTAGCTCAGCA	NOT APPLICABLE
pU6-pegRNA1-PRPH2-828+1G>A-correction	GCTCATTTGGCTCTTCGAGAT	GGCCCAGGGCCTACCTCGAAGAGCC
pU6-pegRNA2-PRPH2-828+1G>A-correction	GCTCATTTGGCTCTTCGAGAT	GGCCCAGGGCCTACCTCGAAGAGCCAA
pU6-pegRNA3-PRPH2-828+1G>A-correction	GCTCATTTGGCTCTTCGAGAT	GGCCCAGGGCCTACCTCGAAGAGCCAAATG
pU6-pegRNA4-PRPH2-828+1G>A-correction	GCTCATTTGGCTCTTCGAGAT	CCCAGCTGGCCCAGGGCCTACCTCGAAGAGCC
pU6-pegRNA5-PRPH2-828+1G>A-correction	GCTCATTTGGCTCTTCGAGAT	CCCAGCTGGCCCAGGGCCTACCTCGAAGAGCCAA
pU6-pegRNA6-PRPH2-828+1G>A-correction	GCTCATTTGGCTCTTCGAGAT	CCCAGCTGGCCCAGGGCCTACCTCGAAGAGCCAAATG
pU6-nsgRNA1-PRPH2-828+1G>A-correction	GCCTTACCCTCTACCCCCAGC	NOT APPLICABLE
pU6-nsgRNA2-PRPH2-828+1G>A-correction	GAGGAGCGTGACGACACCCA	NOT APPLICABLE
pU6-nsgRNA3-PRPH2-828+1G>A-correction	GTTAGACCCAAATGGGACCGG	NOT APPLICABLE
pU6-nsgRNA4-PRPH2-828+1G>A-correction	GACAGGTTGAGCTCCTCCGTC	NOT APPLICABLE

Table S2 – Primer sequences employed for amplifying pegRNA and nsgRNA in the cloning of all-in-one constructs

All-in-one Type	Forward primer (5'-3')	Reverse primer (5'-3')
T1 pU6-pegRNA-HEK3+1CTTins	GCGCTGCTTCGATTTACCTGGAGATGACACGGGT	CTGGCCCGTACATCGTTCG
T1 p7SK-nsgRNA-HEK3	CGATGTACGGGCCAGTTTTGCGCTGCTTCGAGCG	CCCGGGAAGTTATTTGTGTCATCTCCAGGTCCACG
T2 pU6-pegRNA-HEK3+1CTTins	GCGCTGCTTCGATTTACCTGGAGATGACACGGGT	CTGGCCCGTACATCGTTCG
T2 p7SK-nsgRNA-HEK3	CCCGGGAAGTTATTTTGCGCTGCTTCGAGCG	CGATGTACGGGCCAGGTGTCATCTCCAGGTCCACG
T3 pU6-pegRNA-HEK3+1CTTins	ACCTGGAGATGACACGGGT	GCGCTGCTTCGATTTCTGGCCCGTACATCGTTCG
T3 p7SK-nsgRNA-HEK3	CCCGGGAAGTTATTTTGCGCTGCTTCGAGCG	GTGTCATCTCCAGGTGTCATCTCCAGGTCCACG

T4 pU6-pegRNA-HEK3+1CTTins	ACCTGGAGATGACACGGGT	GCGCTGCTTCGATTTCTGGCCCGTAC ATCGTTCG
T4 p7SK-nsgRNA-HEK3	GTGTCATCTCCAGGTTTTGCGCTGCTT CGAGCG	CCCGGGAAGTTATTTGTGTCATCTCC AGGTCCACG
T5 pU6-pegRNA-HEK3+1CTTins	GCAGGAAAGAACATGACCTGGAGAT GACACGGGT	CTGTCGCCGCACATGCTGGCCCGTAC ATCGTTCG
T6 p7SK-nsgRNA-HEK3	GCAGGAAAGAACATGTTTTGCGCTGC TTCGAGCG	CTGTCGCCGCACATGTGTCATCTCCA GGTCCACG
pU6-pegRNA-HEK3	GCGCTGCTTCGATTTACCTGGAGATG ACACGGGT	CTGGCCCGTACATCGTTCG
p7SK-nsgRNA-HEK3	CGATGTACGGGCCAGTTTTGCGCTGC TTCGAGCG	CCCGGGAAGTTATTTGTGTCATCTCC AGGTCCACG
pU6-pegRNA-FANCF	GCGCTGCTTCGATTTACCTGGAGATG ACACGGGT	CTGGCCCGTACATCGTTCG
p7SK-nsgRNA-FANCF	CGATGTACGGGCCAGTTTTGCGCTGC TTCGAGCG	CCCGGGAAGTTATTTGTGTCATCTCC AGGTCCACG
pU6-pegRNA-PRPH2-828+1G>A-knock-in	GCGCTGCTTCGATTTACCTGGAGATG ACACGGGT	CTGGCCCGTACATCGTTCG
p7SK-nsgRNA-PRPH2-828+1G>A-knock-in	CGATGTACGGGCCAGTTTTGCGCTGC TTCGAGCG	CCCGGGAAGTTATTTGTGTCATCTCC AGGTCCACG
pU6-pegRNA2-PRPH2-828+1G>A-correction	GCGCTGCTTCGATTTGATCCG GTACCAAGGTCGGG	CTGGCCCGTACATCGTACCTC GAGCGGCCCAA
pU6-nsgRNA3-PRPH2-828+1G>A-correction	CGATGTACGGGCCAGACCAAG GTCGGGCAGGAA	CCCGGGAAGTTATTCGAGCG GCCCAAGCTTA

Table S3 – Primers used for genomic amplification

**Target**	**Forward primer (5'-3')**	**Reverse primer (5'-3')**
PRPH2	CACCAACAACTCAGCACACTAC	TCCACTGAAGGCTGTTTCCAA
PRPH2-NGS-Barcode1	ACACTCTTTCCCTACACGACGCTCTTCCG ATCTGGAACACCAGACGGAGGAGCTCA	GACTGGAGTTCAGACGTGTGCTCTTCCGATCTCA GAGAGGGAGGCATGCTCTCCA
PRPH2-NGS-Barcode2	ACACTCTTTCCCTACACGACGCTCTTCCG ATCTACCACACCAGACGGAGGAGCTCA	GACTGGAGTTCAGACGTGTGCTCTTCCGATCTTCA GGAGGGAGGCATGCTCTCCA
PRPH2-NGS-Barcode3	ACACTCTTTCCCTACACGACGCTCTTCCG ATCTATGGCACCAGACGGAGGAGCTCA	GACTGGAGTTCAGACGTGTGCTCTTCCGATCTGA TGGAGGGAGGCATGCTCTCCA
PRPH2-NGS-Barcode4	ACACTCTTTCCCTACACGACGCTCTTCCG ATCTCTACCACCAGACGGAGGAGCTCA	GACTGGAGTTCAGACGTGTGCTCTTCCGATCTTAC CGAGGGAGGCATGCTCTCCA
PRPH2-NGS-Barcode5	ACACTCTTTCCCTACACGACGCTCTTCCG ATCTAGTCCACCAGACGGAGGAGCTCA	GACTGGAGTTCAGACGTGTGCTCTTCCGATCTTG GTGAGGGAGGCATGCTCTCCA
PRPH2-NGS-Barcode6	ACACTCTTTCCCTACACGACGCTCTTCCG ATCTGTCTCACCAGACGGAGGAGCTCA	GACTGGAGTTCAGACGTGTGCTCTTCCGATCTCCT TGAGGGAGGCATGCTCTCCA
FANCF	CAGTACGCAGAGAGTCGCC	CGACCAAAGCGCCGATGG
FANCF-NGS-Barcode1	ACACTCTTTCCCTACACGACGCTCTTCCG ATCTGGAAGAACAGTACGCAGAGAGTC GCC	GACTGGAGTTCAGACGTGTGCTCTTCCGATCTGA CGGAACGACCAAAGCGCCGATGG

FANCF-NGS-Barcode2	ACACTCTTTCCCTACACGACGCTCTTCCG ATCTCAGAGAACAGTACGCAGAGAGTCG CC	GACTGGAGTTCAGACGTGTGCTCTTCCGATCTAG TGGAACGACCAAAGCGCCGATGG
FANCF-NGS-Barcode3	ACACTCTTTCCCTACACGACGCTCTTCCG ATCTACCAGAACAGTACGCAGAGAGTCG CC	GACTGGAGTTCAGACGTGTGCTCTTCCGATCTTG GCGAACGACCAAAGCGCCGATGG
FANCF-NGS-Barcode4	ACACTCTTTCCCTACACGACGCTCTTCCG ATCTCCAGGAACAGTACGCAGAGAGTCG CC	GACTGGAGTTCAGACGTGTGCTCTTCCGATCTCTC CGAACGACCAAAGCGCCGATGG
HEK3	ATGTGGGCTGCCTAGAAAGG	GCCCAGCCAAACTTGTCAACC
HEK3-NGS-Barcode1	ACACTCTTTCCCTACACGACGCTCTTCCG ATCTGGAAGAAATGTGGGCTGCCTAGAA AGG	GACTGGAGTTCAGACGTGTGCTCTTCCGATCTGA CGGAAGCCCAGCCAAACTTGTCAACC
HEK3-NGS-Barcode2	ACACTCTTTCCCTACACGACGCTCTTCCG ATCTCAGAGAAATGTGGGCTGCCTAGAA AGG	GACTGGAGTTCAGACGTGTGCTCTTCCGATCTAG TGGAAGCCCAGCCAAACTTGTCAACC
HEK3-NGS-Barcode3	ACACTCTTTCCCTACACGACGCTCTTCCG ATCTACCAGAAATGTGGGCTGCCTAGAA AGG	GACTGGAGTTCAGACGTGTGCTCTTCCGATCTTG GCGAAGCCCAGCCAAACTTGTCAACC
HEK3-NGS-Barcode4	ACACTCTTTCCCTACACGACGCTCTTCCG ATCTCCAGGAAATGTGGGCTGCCTAGAA AGG	GACTGGAGTTCAGACGTGTGCTCTTCCGATCTCTC CGAAGCCCAGCCAAACTTGTCAACC
KI-pegRNA-OT1-CHRNB4	ACGAGGTGGTGCTTTCCCT	CACTTGGCAAGGGATCGGG
KI-pegRNA-OT2-No coding	AGCAGTAATGCTAGGGGCT	TAGGGCTTCTAGGCCTGGC
KI-pegRNA-OT3-NCAM2	TGGTGCAGCCAGAAAAAGAATGC	TTTGGCCAAGGTAAGGACATTGG
KI-pegRNA-OT4-ZFHX3	TGGGACTGGAAGGTCACCC	CACATGCGATGGAGGGTGC
KI-pegRNA-OT5-IFT43	TGTGGGAACTTGGTTTCGGAC	CATTGCAAGGGAGGCAGAGT
KI-pegRNA-OT6-No coding	CCACAATTACTTCTGCACCAACC	CCTGCTGCACTGAGTTACCA
KI-nsgRNA-OT1-DMTN	AACTACCACCTCTAGGCAAATGG	AGGTCGTAATCTGTTGGCAACAG
KI-nsgRNA-OT2-No coding	GAGGAACAGGGGCATGCAC	ACCCAGCCACTTGTTCCCT
KI-nsgRNA-OT3-ZNF286A/TBC1D26	GCCTTACACAGCCATGCCA	AGCCGTCTGACTTGAGGTG
KI-nsgRNA-OT4-TBC1D27P	GGGAGGCAGGAGGACACTC	ACAGTCATGCCAGGGGACA
KI-nsgRNA-OT5-RGS3	GCTGACAGGACACAGCCTG	CGCAGCAAAAGCCAGGAGA
KI-nsgRNA-OT6-UBA1	ACAGGAAGGCGTGCTCACT	AGGCCCAGTGGGTTGGAAT
KI-nsgRNA-OT7-No coding	GGGAAGGTGCTCAGAAGCC	CTTGGGCTCCCCTTTGCTG
KI-nsgRNA-OT8-SWAP70	TGATTCAGCAGATCGTTAGACTGG	GTCACCCTAACCAAGCATTTATCA
KI-nsgRNA-OT9-RAB6B	CTGTCCGGTGGGGATGAGT	TGTGAGGTGTGATGGGGTTG

*OT stands for off targeting

Table S4 – Primers and probes used for real-time PCR

**Target**	**Forward primer (5'-3')**	**Reverse primer (5'-3')**	**Probes (5'-3')**
Total PRPH2 Transcript	GAACTCCATGGGTGTCGTC	CCAGCGACGTCTGTAGGTA	**5HEX/** ACGCTCCTC **/ZEN/** ATTTGGCTCTTCGAG **/3IABkFQ/**
Canonical PRPH2 Transcript	GAACTCCATGGGTGTCGTC	CCAGCGACGTCTGTAGGTA	**5SUN/** TTGGCTCTT **/ZEN/** CGAGGTGACCATTACAA **/3IABkFQ/**
Mutant PRPH2 Transcript	GAACTCCATGGGTGTCGTC	CCAGCGACGTCTGTAGGTA	**56-FAM/** TTGGCTCTT **/ZEN/** CGAGATAGGCCCTGG **/3IABkFQ/**
β-actin	ACAGAGCCTCGCCTTTG	CCTTGCACATGCCGGAG	**56-FAM/** TCATCCATG **/ZEN/** GTGAGCTGGCGG **/3IABkFQ/**

Supplemental Figures

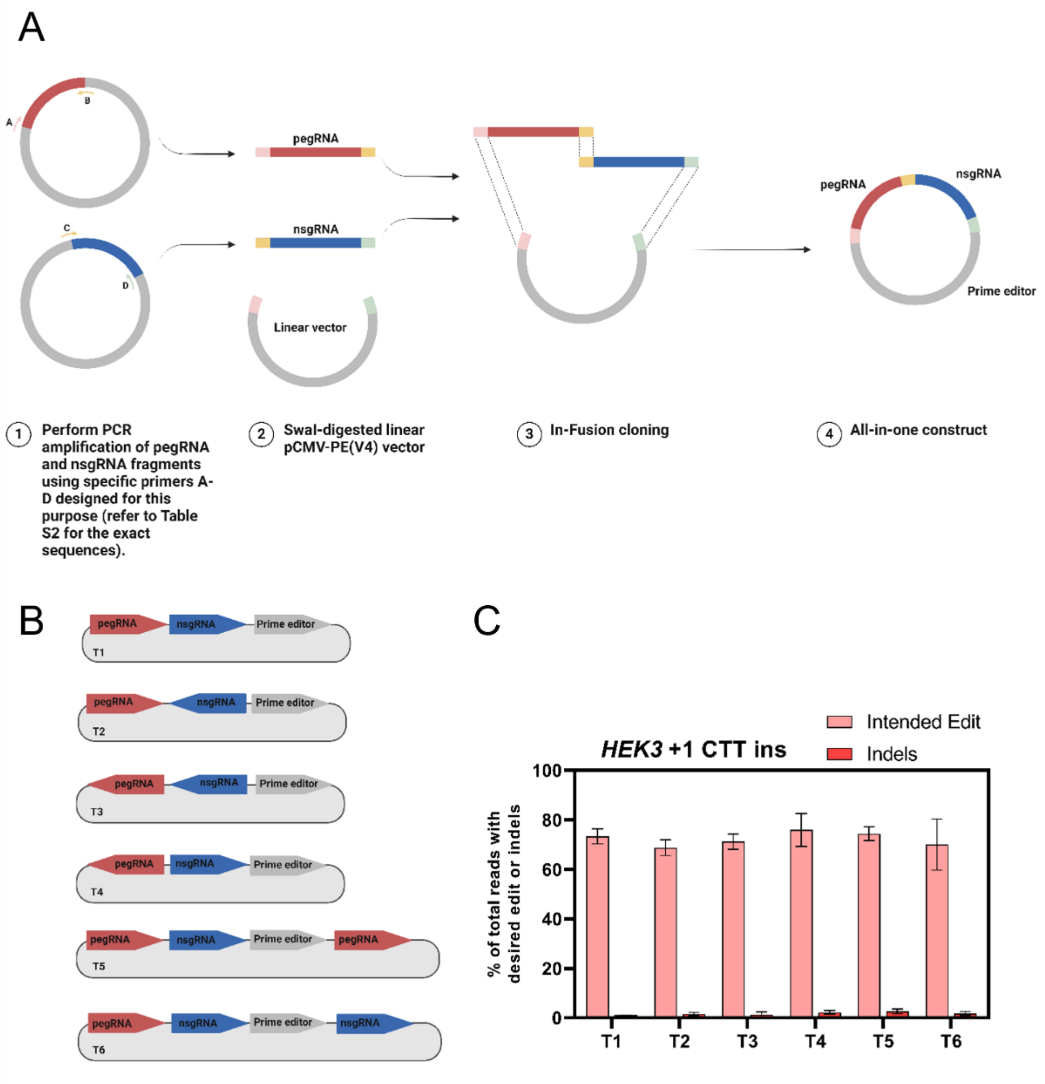

Figure S1. Development of an All-in-One Prime Editing System. (A) Overview of the all-in-one assembly protocol, which includes four steps: Step 1 involves PCR amplification of the pegRNA and nsgRNA fragments. Step 2 consists of linearizing the pCMV-PE(V4) vector using the *SwaI* restriction enzyme. Step 3 is the infusion cloning process that leads to the generation of the all-in-one construct in Step 4. (B) Designs for our all-in-one system. In the T1-T4 designs, we modified the orientation of the pegRNA, nsgRNA, and their respective promoters. In T5 and T6, we enhanced the expression levels of pegRNA and nsgRNA, respectively. (C) PE editing efficiency of T1-T6 all-in-one designs for installing the HEK3 +1CTTins in HEK293T cells. Data are presented as mean ± SD (n=2). Schematics created using Biorender.

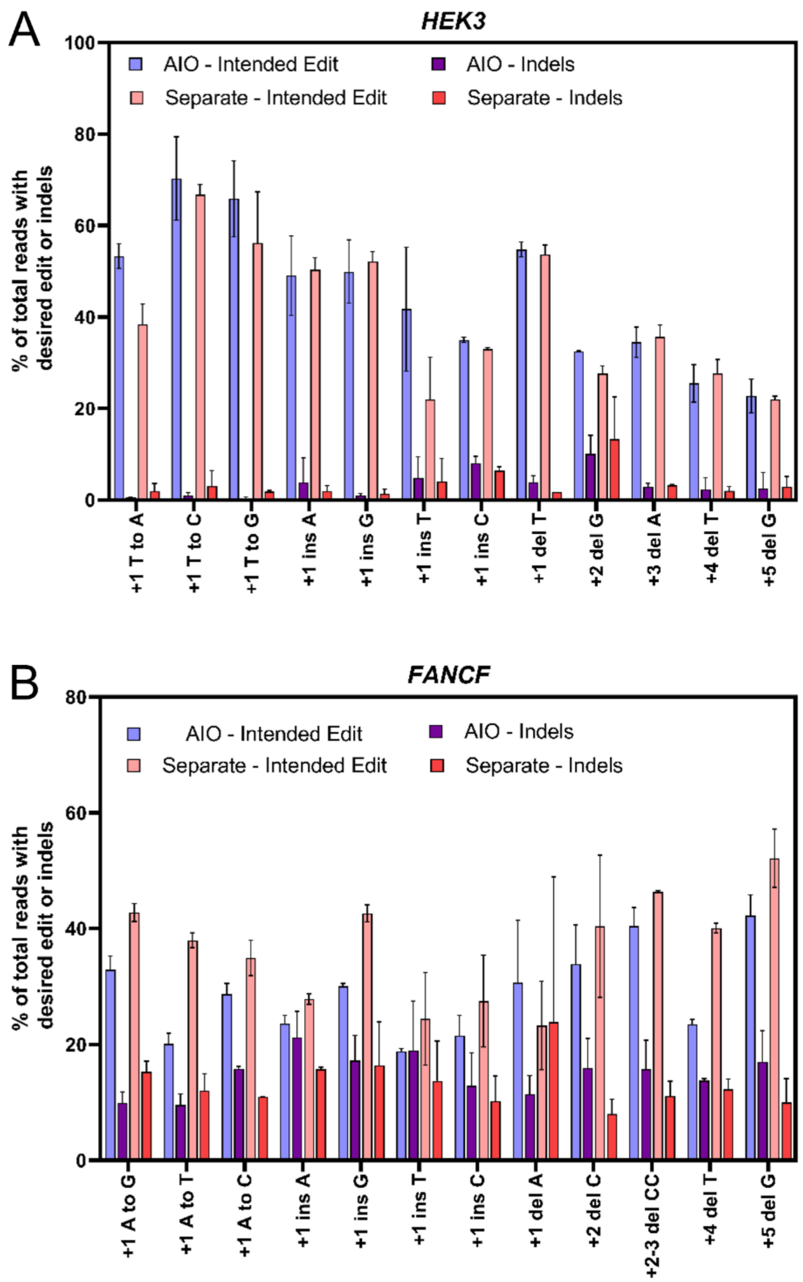

Figure S2. All-in-One PE Demonstrates Comparable Editing Efficiency to Separate Systems. Comparison of separate and all-in-one systems for different types of edits in the (A) *HEK3* and (B) *FANCF* locus. Data are presented as mean ± SD (n=2).

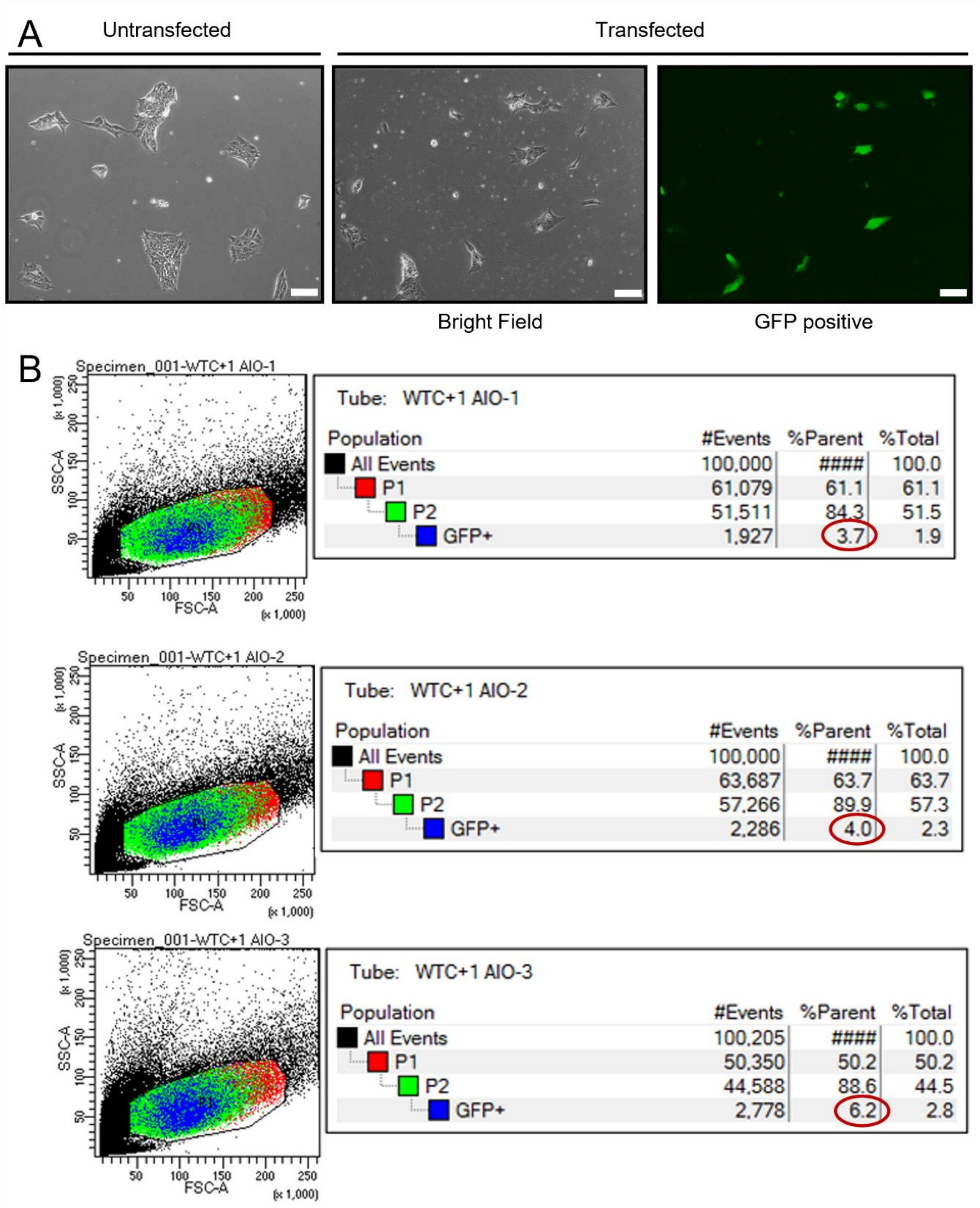

Figure S3. Delivery of the All-in-One PE System for Introducing the *PRPH2* c.828+1G>A Variant in hiPSCs. (A) Images of untransfected hiPSCs and hiPSCs transfected using Lipofectamine™ Stem. Representative images are shown, with a scale bar indicating 100 µm. (B) Quantification of GFP-positive hiPSCs (red circles) sorted by FACS, presented as triplicate measurements.

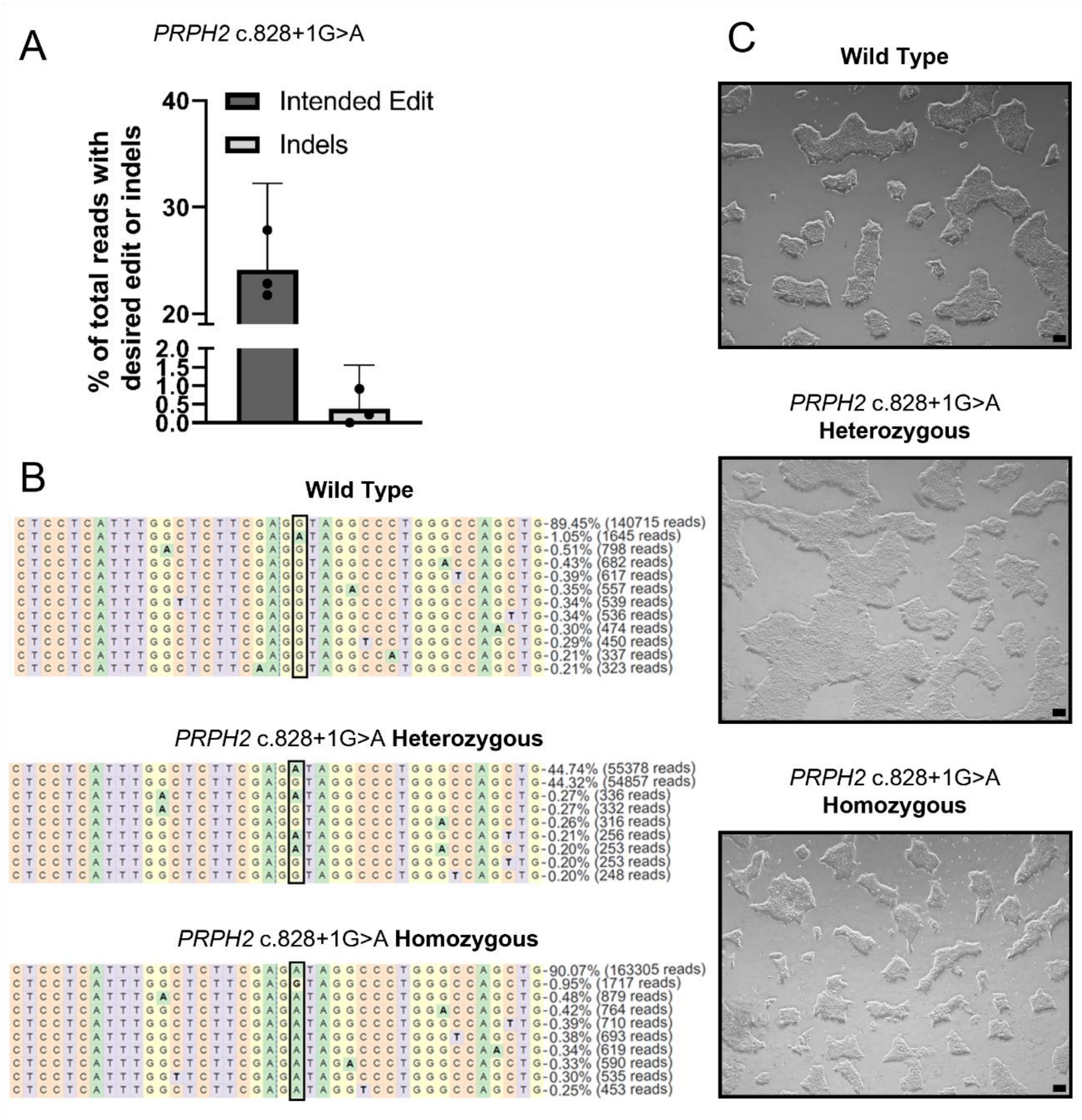

Figure S4. Efficient Installation of the *PRPH2* c.828+1G>A Variant in hiPSC. (A) Quantification of the percentage of total reads containing the desired edits or indels, assessed by next-generation sequencing from pooled GFP-positive cells at 6 days post-transfection. Data are presented as mean ± 95% CI (n=3). (B) Next-generation sequencing validation confirming the successful introduction of the *PRPH2* c.828+1G>A variant in WTC11 hiPSCs, detected in both heterozygous and homozygous states. (C) Bright-field images showing the normal cellular morphology of *PRPH2* c.828+1G>A wild-type, heterozygous, and homozygous hiPSCs. Representative images are shown, with a scale bar indicating 100 μm.

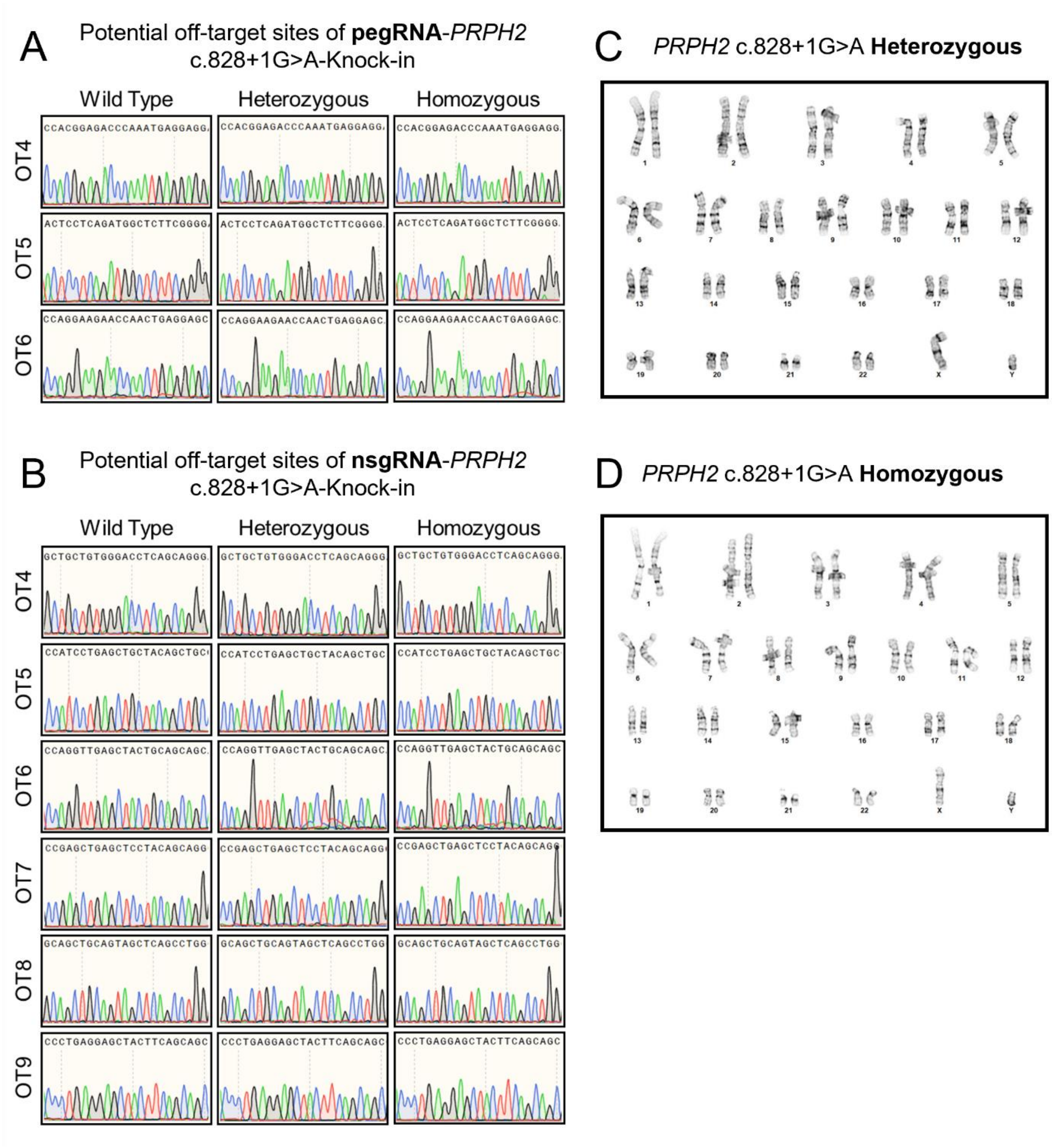

Figure S5. Prime Editing for the *PRPH2* c.828+1G>A Variant in hiPSC Shows no Off-Target Effects or Chromosomal Abnormalities. (A-B) Dideoxy sequencing traces indicate no off-target effects in heterozygous and homozygous clones at (A) Remaining three potential off-target sites associated with pegRNA-*PRPH2* c.828+1G>A knock-in. (B) Remaining six potential off-target sites associated with nsgRNA-*PRPH2* c.828+1G>A knock-in. (C-D) Karyotype analysis shows no chromosomal abnormalities in edited clones: (C) heterozygous and (D) homozygous.

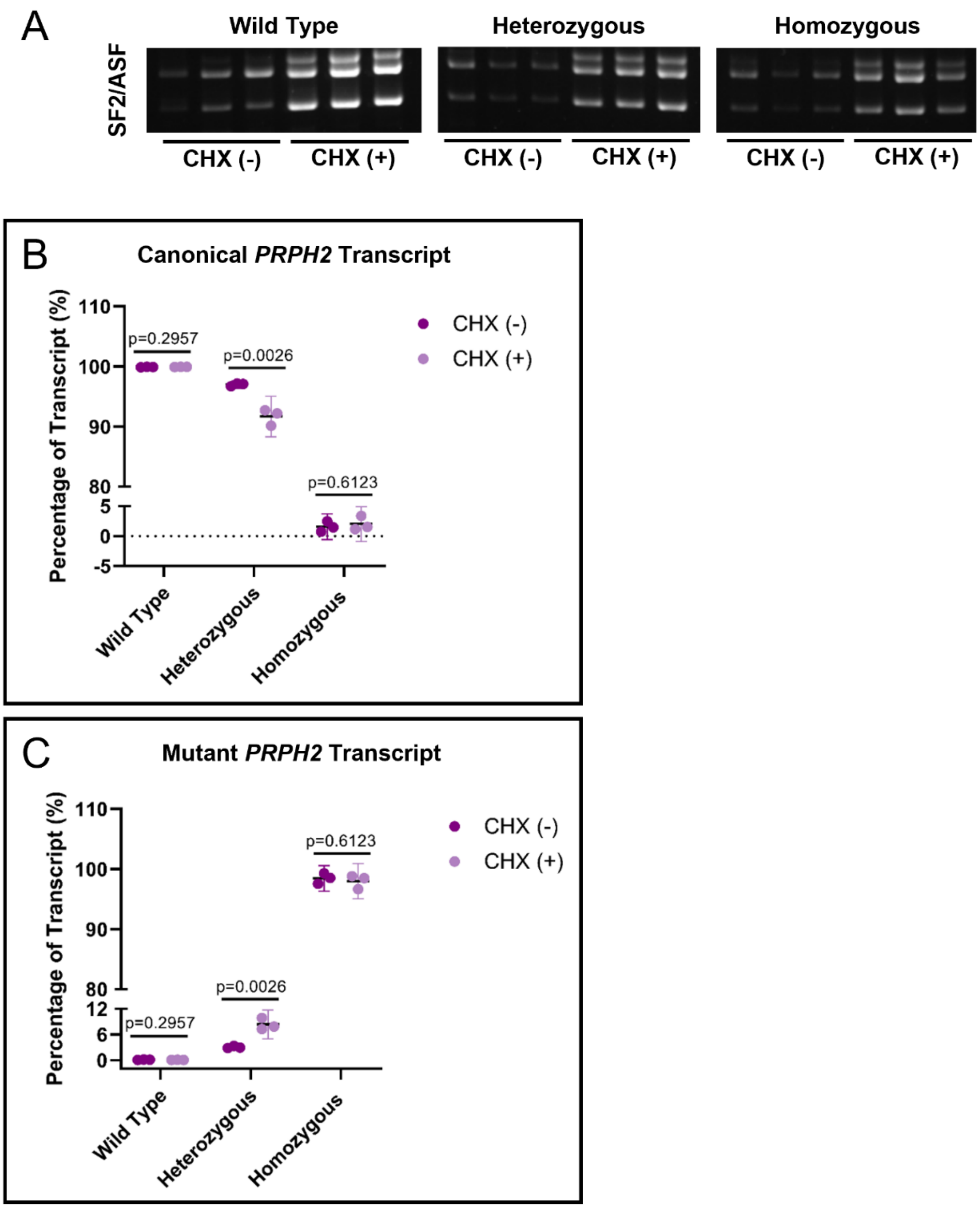

Figure S6. Cycloheximide (CHX) Prevents Nonsense-mediated Decay of the Mutant *PRPH2* Transcript in the *PRPH2* c.828+1G>A Heterozygous hiPSC. (A) Representative agarose gel confirming increased SF2/ASF transcript expression as a positive control for CHX treatment. (B-C) Quantification of *PRPH2* transcript levels by real-time PCR. (B) Canonical *PRPH2* transcript levels, and (C) mutant *PRPH2* transcript levels. Data are presented as mean ± 95% CI (n=3).

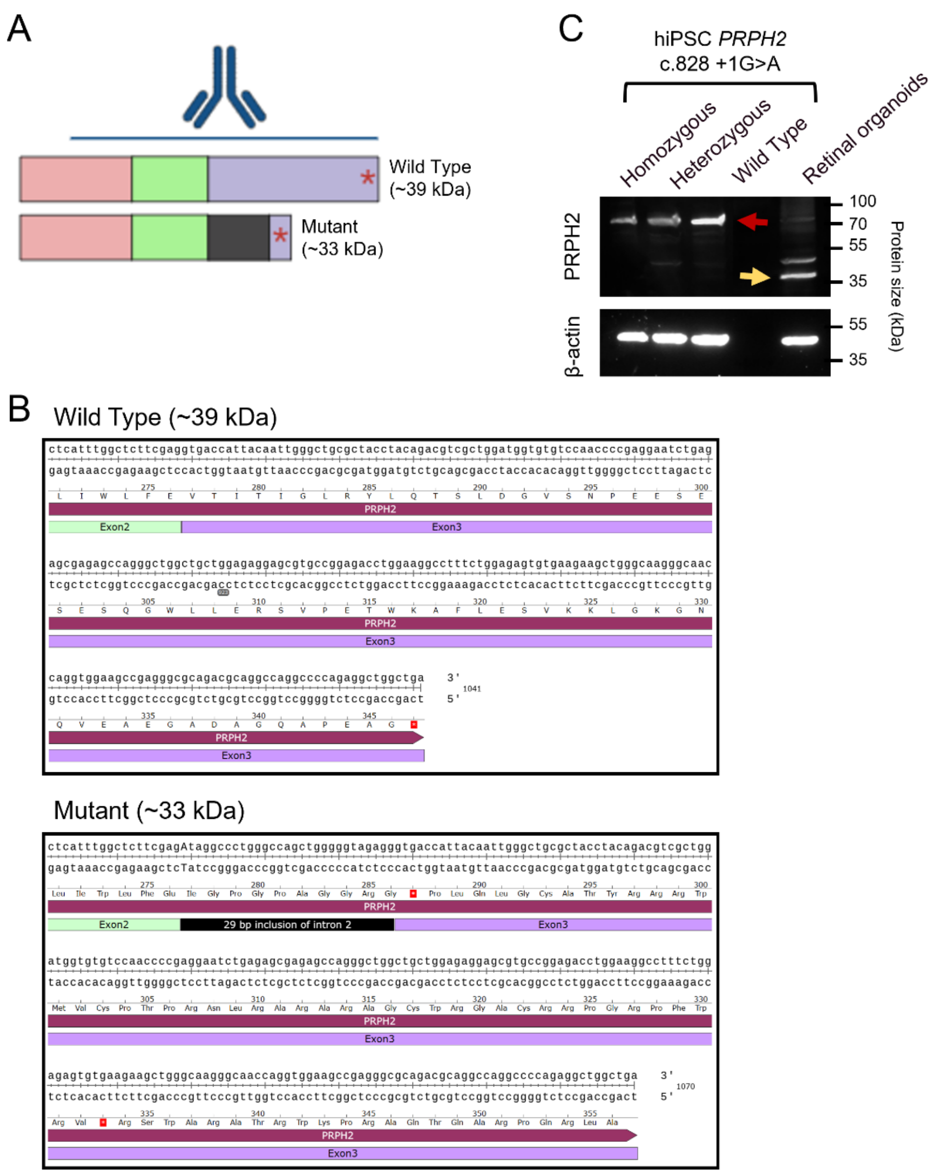

Figure S7. PRPH2 Protein Presence in hiPSCs and Retinal Organoids. (A) Schematic representation of the PRPH2 protein structure highlighting the antibody binding epitope in the wild-type and mutant forms. Red asterisks illustrate the stop-codon position. (B) SnapGene analysis predicts that the *PRPH2* c.828+1G>A mutation results in a truncated protein product. (C) Western blot analysis showing PRPH2 protein presence in hiPSCs and wild type retinal organoids (DD225). In hiPSCs, PRPH2 is detected predominantly as dimeric form (red arrow), whereas in retinal organoids, the monomeric form is more prominent (yellow arrow). Schematic created using Biorender.

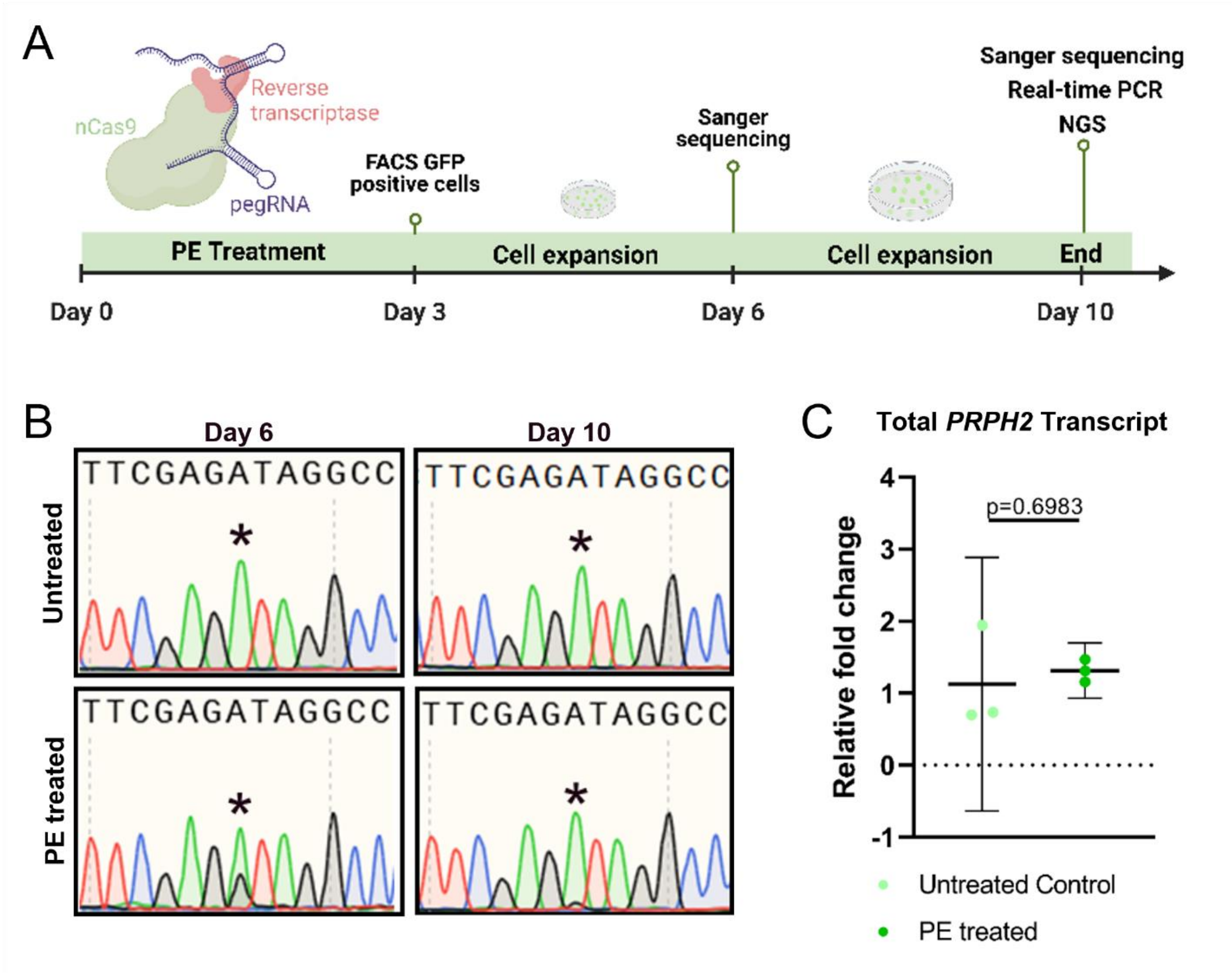

Figure S8. Prime Editing Corrects the *PRPH2* c.828+1G>A Mutation in hiPSCs. (A) Illustration of the experimental design (B) Dideoxy traces demonstrating the correction of homozygous *PRPH2* c.828+1G>A hiPSCs at 6 and 10 days following prime editing treatment. (C) Quantification of total *PRPH2* transcripts by real-time PCR. Data is expressed as mean ± 95% CI (n=3). Untreated Control = c.828+1G>A homozygous line, PE treated = the treated c.828+1G>A homozygous line that shows wild type genotype. Schematic created using Biorender.
