# Supplementary material for: Prime editing for the investigation of aberrant splicing defect associated with a pathogenic PRPH2 variant
Source: Mol Ther Nucleic Acids. 2025 Oct 13;36(4):102740. doi: 10.1016/j.omtn.2025.102740 (PMC12594906; doi:10.1016/j.omtn.2025.102740)
Supplement: Document S2. Article plus Supplemental information [file mmc2.pdf]

Molecular Therapy
Nucleic Acids
Original Article

Prime editing for the investigation of aberrant splicing defect associated with a pathogenic *PRPH2* variant

Bruna Lopes da Costa,[1,2,3] Kyle M. Helms,[4,5] Keith Theodore,[3] Yi-Ting Tsai,[1,2] Salvatore Marco Caruso,[1,2] Siyuan Liu,[1,2] Jose Ronaldo Lima de Carvalho,[3] Nicholas D. Nolan,[1,2] Saleha Tahir,[3] Christopher D. Makinson,[4,5,6] Stephen H. Tsang,[1,2,7,8,9] and Peter M.J. Quinn[3]

[1]Jonas Children's Vision Care (JCVC) and Barbara & Donald Jonas Stem Cell Laboratory, New York-Presbyterian Hospital, New York, NY 10032, USA; [2]Department of Biomedical Engineering, Columbia University, New York, NY 10027, USA; [3]FM Kirby Center for Molecular Ophthalmology, Department of Ophthalmology, University of Pennsylvania Perelman School of Medicine, Philadelphia, PA 19104, USA; [4]Department of Neurology, Columbia University Irving Medical Center, New York, NY 10032, USA; [5]Center for Translational Research in Neurodevelopmental Disease, Columbia University Irving Medical Center, New York, NY 10032, USA; [6]Department of Neuroscience, Columbia University Irving Medical Center, New York, NY 10032, USA; [7]Department of Ophthalmology, Columbia University Irving Medical Center, New York, NY 10032, USA; [8]Department of Pathology & Cell Biology, Columbia University Irving Medical Center, New York, NY 10032, USA; [9]Columbia Stem Cell Initiative, Institute of Human Nutrition, Vagelos College of Physicians and Surgeons, Columbia University Irving Medical Center, New York, NY 10032, USA

The human Peripherin 2 (*PRPH2*) gene, essential for the structure and function of photoreceptor outer segments, is implicated in a range of inherited retinal diseases (IRDs). This study focuses on the pathogenic c.828+1G>A *PRPH2* splice site variant. We employed prime editing (PE) technology to install and correct this variant in human induced pluripotent stem cells (hiPSCs). We developed an all-in-one PE construct, featuring a GFP reporter to facilitate the identification of successfully edited clones. The resulting heterozygous and homozygous hiPSC clones exhibited no detectable off-target mutations or karyotype abnormalities. Crucially, we found in hiPSCs and DD50/DD100 precursor hiPSC-derived retinal organoids that the c.828+1G>A *PRPH2* mutation leads to activation of a cryptic splice site and intron retention, forming a mutant transcript. Importantly, correction of the c.828+1G>A *PRPH2* mutation in the homozygous hiPSC clone resulted in the restoration of the canonical *PRPH2* transcript and a reduction of the mutant transcript. Our findings highlight the potential of PE as a precise and safe method for installing and correcting pathogenic *PRPH2* mutations in hiPSCs, paving the way for future genotype-phenotype studies and therapy development for *PRPH2*-mediated IRDs.

INTRODUCTION

The human Peripherin-2 (*PRPH2*) gene, also known as retinal degeneration slow (*RDS*), encodes a 346 amino-acid-long tetraspanin protein that localizes to the rod and cone photoreceptor outer segments and is crucial for forming, maintaining, and renewing the outer segment discs.[1,2] Mutations in *PRPH2* lead to the disorganization or absence of photoreceptor outer segments, causing disabling inherited retinal diseases (IRDs). *PRPH2* mutations exhibit high phenotypic variability, with a total of 367 variants that have been reported to date on the Leiden Open Variation Database (LOVD, accessed 10/02/24). The gene has been linked to a variety of clinical manifestations including autosomal dominant retinitis pigmentosa (adRP), adult vitelliform macular dystrophy, pattern macular dystrophy (PD), fundus-flavimaculatus-like dystrophy, central areolar choroidal dystrophy (CACD), and cone-rod dystrophy (CRD).[1,2] In the United States, *PRPH2* mutations occur in about 1 in 54,062 individuals, and they contribute to 5%–10% of adRP cases worldwide.[3,4] Despite the large number of mutations identified, there is still no clear genotype-phenotype correlation, and, to date, there is no treatment available for *PRPH2*-mediated IRD patients.

A defining feature of *PRPH2*-mediated IRDs is their remarkable phenotypic variability, which presents a unique challenge for both disease modeling and therapeutic development. Experimental studies suggest that PRPH2 is highly dosage-sensitive, with rods more vulnerable to haploinsufficiency and cones more affected by toxic, dominant-negative mutations.[5–13] This differential sensitivity necessitates a nuanced approach to gene therapy—one that considers the distinct functional requirements of PRPH2 in each photoreceptor subtype. Modeling *PRPH2*-mediated IRDs is further complicated by the rarity of these conditions. Patient-derived biological material is difficult to obtain, often requiring invasive procedures and costly derivation of human induced pluripotent stem cells

Received 28 October 2024; accepted 9 October 2025;
https://doi.org/10.1016/j.omtn.2025.102740.

Correspondence: Peter M.J. Quinn, Ph.D., FM Kirby Center for Molecular Ophthalmology, Department of Ophthalmology, University of Pennsylvania Perelman School of Medicine, Philadelphia, PA 19104, USA.
E-mail: peter.quinn1@pennmedicine.upenn.edu

© 2025 The Author(s).
Published by Elsevier Inc. on behalf of The American Society of Gene and Cell Therapy.
This is an open access article under the CC BY license (http://creativecommons.org/licenses/by/4.0/).

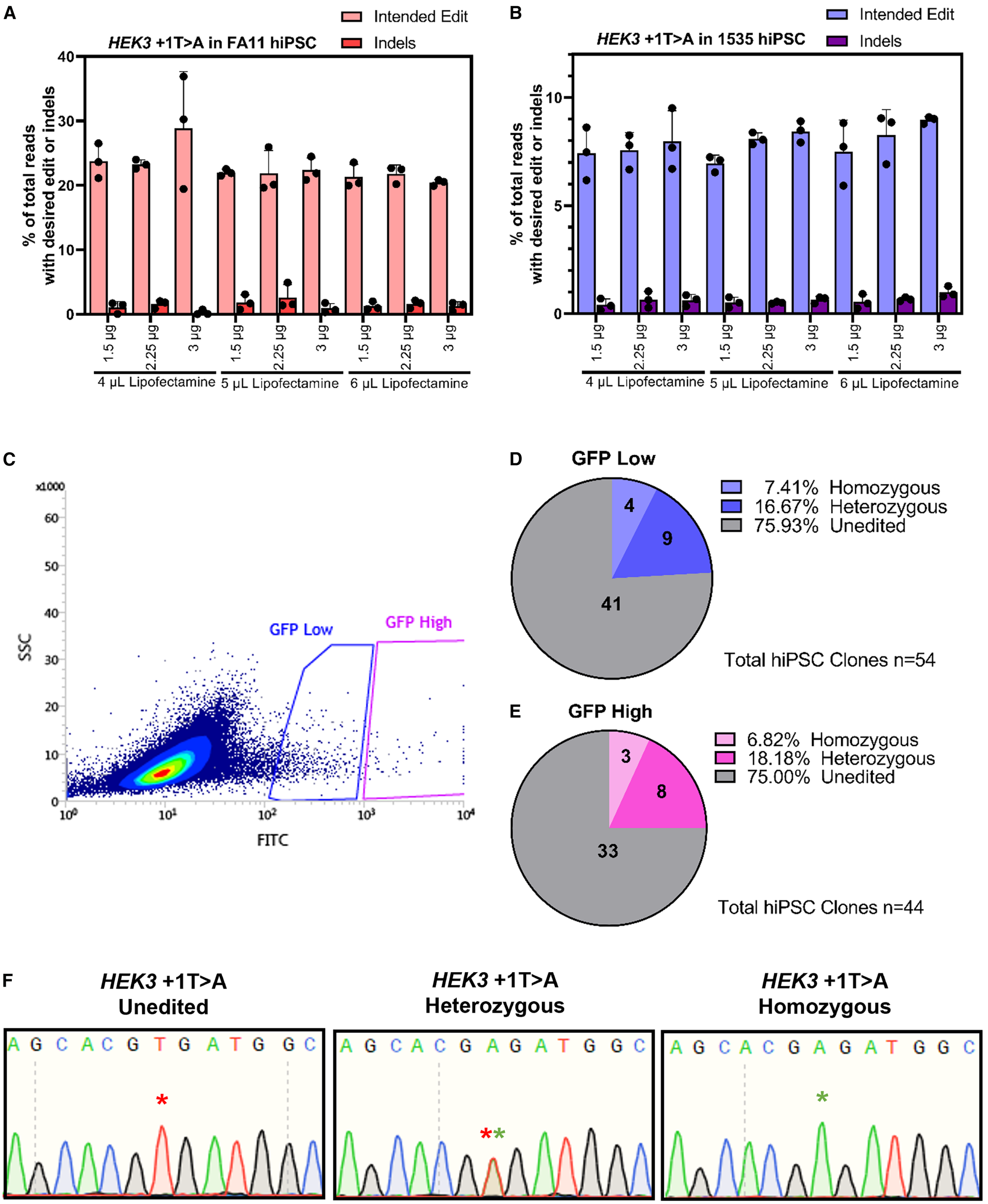

(legend on next page)

(hiPSCs).[14] Even when successful, patient-derived hiPSCs frequently exhibit chromosomal abnormalities, donor- and clone-dependent variability in pluripotency levels, and epigenetic alterations that impair their capacity to generate complex *in vitro* models such as retinal organoids.[15–17] These challenges have led to increasing interest in generating isogenic hiPSC models in established, well-characterized hiPSC lines. These models, which carry engineered disease-relevant mutations on a healthy genetic background, provide a powerful platform for mechanistic studies and preclinical therapeutic testing.

Genome editing using clustered regularly interspaced short palindromic repeats (CRISPR)/CRISPR-associated (Cas) systems has broadly been used to install or correct variants to create isogenically paired hiPSC lines, advancing the understanding of IRD mechanism, including mutations associated with mis-splicing defects, as well as for therapeutic development.[18,19] Prime editing (PE) represents a novel CRISPR/Cas9 technology capable of correcting both transition and transversion mutations, as well as small deletions and insertions, without inducing double-strand breaks (DSBs).[20–22] In the PE approach, a PE guide RNA (pegRNA) is employed alongside a prime editor, composed of the Cas9 nickase fused with an optimized reverse transcriptase. The pegRNA includes a spacer, a primer binding sequence (PBS), and an reverse transcription template (RTT) encoding the desired edit. This combination of the engineered prime editor and pegRNA constitutes the prime editing 2 strategy (PE2). In the PE3 strategy, an additional nicking single guide RNA (nsgRNA) is utilized to nick the non-edited strand, directing DNA repair to utilize the edited strand as a template, thereby enhancing editing efficiency.[20–22] This method offers precise correction across a broad spectrum of mutations, yet to date, has been understudied for correcting human mutations associated with IRDs, including variants in *PRPH2*.[14,21]

In this study, we employed a PE strategy to install and correct the rare *PRPH2* c.828+1G>A variant in hiPSCs. The *PRPH2* c.828+1G>A variant is one of several c.828+ (formerly annotated as IVS2+) splice site mutations, including the second most commonly reported *PRPH2* mutation c.828 + 3A>T (LOVD, accessed 06/26/24).[2,23–28] We designed an all-in-one PE construct incorporating a GFP reporter and optimized transfection conditions to enhance the isolation of edited clones. PE facilitated the generation of edited *PRPH2* c.828+1G>A knock-in clones without detectable genomic alterations at predicted off-target sites, as confirmed by dideoxy sequencing. Importantly, the gene editing process did not affect cell karyotype or the expression of pluripotency markers. Examination of *PRPH2* c.828+1G>A hiPSCs and DD50/DD100 precursor retinal organoids highlighted the presence of abnormal splicing due to activation of a cryptic splice site, which was then corrected using PE genome editing technology. This mutant transcript was previously detected in peripheral white blood cells collected from *PRPH2* patients with the prevalent c.828+3A>T variant.[25] Our findings underscore PE as a tool for generating the *PRPH2* c.828+1G>A variant, thereby facilitating future genotype-phenotype studies that will also inform us about the mechanisms of retinal degeneration for the other *PRPH2* c.828 splice site mutations that activate the same cryptic splice site. Moreover, PE demonstrates promising potential for correcting this mutation, offering insights into its future therapeutic application.

RESULTS

All-in-one prime editing system efficiently generates edited hiPSCs clones

We investigated a PE strategy for installation and correction of the *PRPH2* c.828+1G>A splicing variant in hiPSCs. For a PE3 approach, the prime editor, pegRNA, and nsgRNA must be delivered to the same cell to achieve effective editing.[20] To aid this process, we developed an all-in-one PE construct with a GFP reporter, which enables the screening of transfected cells by selecting GFP-positive ones (Figure S1A).

Transcriptional interference is a phenomenon where one transcriptional process is suppressed by another.[29] It is a common event in nature to regulate gene expression.[30,31] In brief, the strength of a specific promoter can be affected by the orientation of other promoters as well as by the open reading frame properties, including position and orientation.[32,33] To account for this, we tested six distinct designs for our all-in-one system. In T1–T4 designs, we altered the orientation of the pegRNA, nsgRNA, and their respective promoters, while in T5 and T6, we increased the expression of pegRNA and nsgRNA, respectively (Figure S1B). The insertion efficiency of CTT at position +1 of the *HEK3* locus in HEK293T cells was similar across all designs, indicating no transcriptional interference affecting the expression of any PE components in our all-in-one system. Furthermore, increasing the expression of pegRNA or nsgRNA did not enhance editing efficiency (Figure S1C). As a result, we opted to proceed with the T1 design for future experiments.

Importantly, the all-in-one PE system demonstrated similar editing efficiency compared to separate plasmid delivery for editing two genomic loci (*HEK3* and *FANCF*) (Figure S2). Due to the known inefficiency of electroporating large plasmids, such as the 12 Kb all-in-one PE,[34] we optimized its transfection using Lipofectamine Stem in two hiPSC lines. We found that varying the amount of DNA and Lipofectamine did not impact editing efficiency (Figures 1A and 1B). Consequently, we proceeded with the lowest amounts of DNA and Lipofectamine for the subsequent experiments. Fluorescence-activated cell

Figure 1. Optimization of all-in-one prime editor in hiPSCs

PE editing efficiency for the introduction of the HEK3 +1T>A variant was assessed following the delivery of varying amounts of DNA and Lipofectamine in (A) FA11 hiPSCs and (B) 1535 hiPSCs. Data presented as mean ± SD (n = 3). (C) Fluorescence-activated cell sorting (FACS) conditions used to separate high and low GFP-expressing cells. (D and E) Efficiency of isolating unedited, heterozygous, and homozygous clones through sorting of (D) low GFP and (E) high GFP hiPSCs. (F) Dideoxy sequencing traces depicting unedited, heterozygous, and homozygous HEK3 +1T>A isolated single clones.

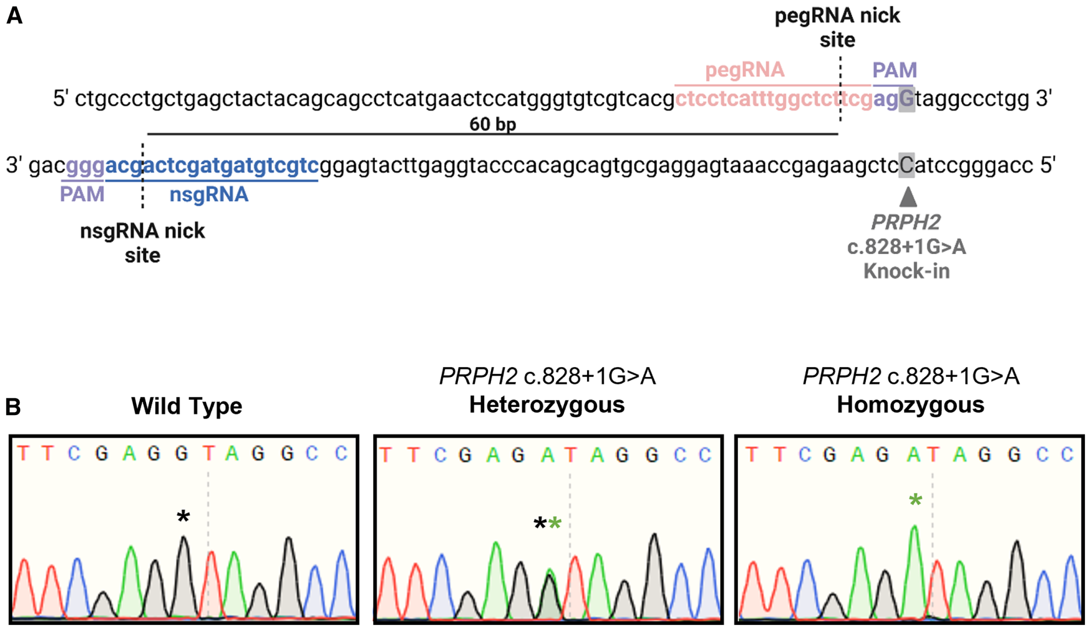

Figure 2. Efficient installation of the *PRPH2* c.828+1G>A variant in hiPSC by prime editing

(A) Prime editing design for knocking in the *PRPH2* c.828+1G>A splice mutant in hiPSCs, indicating the edit's position along with the pegRNA and nsgRNA. (B) Dideoxy sequencing traces showing unedited, heterozygous, and homozygous isolated *PRPH2* c.828+1G>A clones. Schematic created using BioRender.

sorting (FACS) was employed to enrich cells containing all PE components. Interestingly, high GFP expression did not improve the isolation of edited clones compared to low GFP expression, with both populations showing around 24% editing rate, including 7% homozygous and 17% heterozygous edited clones (Figures 1C–1F). The *PRPH2* c.828+1G>A mutation is rare, making the acquisition of patient-derived iPSCs a significant challenge. Using our optimized method, we achieve an editing efficiency of ~24% with indels below 0.5% in pooled GFP-positive cells (Figures S3A, S3B, and S4A). Following this, we successfully generated both heterozygous and homozygous *PRPH2* c.828+1G>A knock-in hiPSC clones (Figures 2A, 2B, S4B, and S4C).

Collectively, these findings indicate that PE is an effective approach for introducing the *PRPH2* c.828+1G>A mutation in hiPSCs.

Prime editing does not result in off-targeting effects, and it does not affect hiPSCs pluripotency

A significant challenge in gene editing is identifying and measuring unintended genomic changes in off-target areas, often caused by the presence of homologous sequences across the genome. We used dideoxy sequencing to investigate the genomic integrity of edited hiPSC clones and assess the precision of the PE approach. We analyzed the genomic regions that matched the spacer sequences of the pegRNA and nsgRNA used to install the *PRPH2* c.828+1G>A variant in hiPSC.

We predicted the off-targets using Cas-OFFinder, permitting up to three mismatches in the DNA sequence relative to the spacer sequences of the pegRNA and nsgRNA. Our analysis revealed 12 potential off-target sites for the pegRNA and 9 for the nsgRNA. Of the 12 off-target sites associated with the pegRNA, 6 belong to the nuclear pore complex-interacting protein B (*NPIPB*) subfamily, which exhibits significant sequence homology, preventing specific amplification by PCR. Nevertheless, since PE is a DSB-free strategy, off-target effects are hypothesized to be minimal. Supporting this hypothesis, we observed no off-target effects in the *PRPH2* c.828+1G>A heterozygous and homozygous clones at the assessed off-target sites (Figures 3A, 3B, S5A, and S5B).

Recent studies have indicated that karyotype abnormalities may be linked to CRISPR/Cas9 genome editing.[35,36] These chromosomal rearrangements—such as deletions, inversions, duplications, and translocations—typically arise when DNA DSBs are formed at two distinct sites, leading to the rejoining of the DNA ends in a different configuration.[37] While PE does not induce DSBs, the concurrent targeting of the genome by pegRNA and nsgRNA in close proximity could potentially lead to DSB formation.[38] With this consideration, we aimed to assess the potential chromosomal aberrations associated with our PE approach. The karyotype analysis revealed that our PE strategy did not lead to any chromosomal aberrations in the isolated *PRPH2* c.828+1G>A heterozygous and homozygous clones (Figures S5C and S5D).

A key feature of hiPSCs is their capacity to proliferate in culture and differentiate into specialized cell types. We investigated whether the PE event impacted the pluripotency of edited hiPSCs. The PE-edited

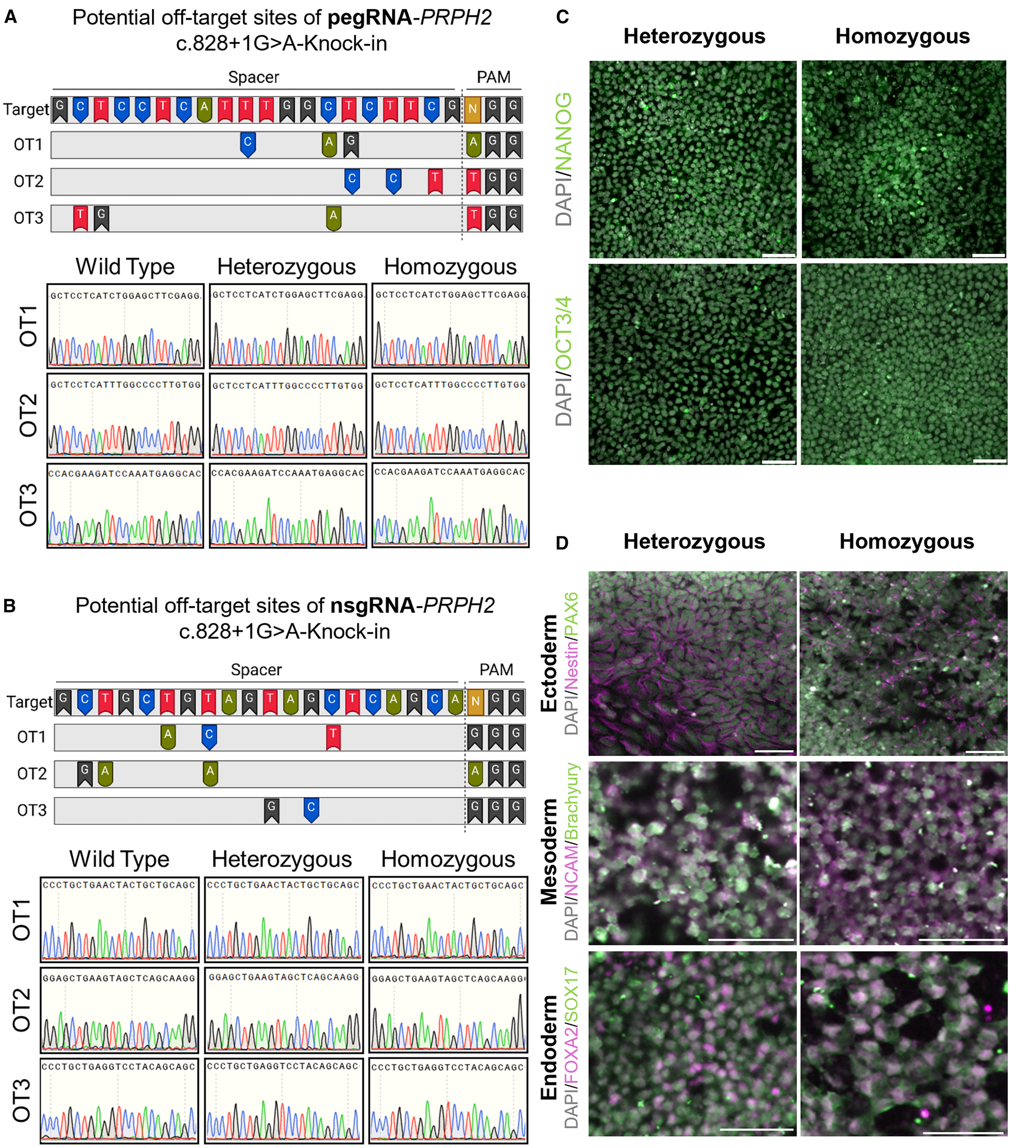

Figure 3. Characterization of heterozygous and homozygous edited clones demonstrates that prime editing events do not induce off-target effects and do not affect cell pluripotency

(A and B) Dideoxy sequencing traces demonstrate no off-target effects in heterozygous and homozygous clones at three potential off-target sites associated with (A) pegRNA-*PRPH2* c.828+1G>A knockin and (B) nsgRNA-*PRPH2* c.828+1G>A knockin. (C) Immunofluorescence images reveal the expression of pluripotency markers

(legend continued on next page)

clones maintained their characteristic hiPSC colony morphology (Figure S4C). Immunofluorescence analysis confirmed that the edited clones expressed the pluripotency markers NANOG and OCT3/4 (Figure 3C), indicating that PE did not affect their pluripotent state. Furthermore, we assessed whether the editing event hindered the ability of the *PRPH2* c.828+1G>A heterozygous and homozygous clones to differentiate into the three germ layers. We subjected the PE-edited clones to ectodermal, mesodermal, and endodermal differentiation and analyzed the expression of Nestin/PAX6, NCAM/Brachyury and FOX2A/SOX17 markers, respectively. Our findings showed that PE did not compromise the ability of these cells to differentiate into the three germ layers (Figure 3D), suggesting that these clones can generate all committed cell types found in human tissues.

The *PRPH2* c.828+1G>A mutation causes aberrant splicing in hiPSCs

Previously, Shankar and colleagues reported an aberrant splicing defect due to the activation of a downstream cryptic splice site, leading to the retention of 29 bp of intron 2 in transcript from peripheral white blood cells of patients with the prevalent *PRPH2* c.828+3A>T mutation.[25] Similarly, we found that the *PRPH2* c.828+1G>A variant caused the same splicing defect (Figures 4A–4D). Using the Netgene2 splice site prediction program, we compared the wild-type *PRPH2* sequence against the *PRPH2* c.828+1G>A sequence, finding complete loss of the predicted canonical splice site (Figure 4A). Subsequently, using cDNA from the wild-type and *PRPH2* c.828+1G>A homozygous knockin hiPSC lines, we amplified a PCR product spanning exons 2 and 3 of *PRPH2*. We found a higher-molecular-weight product amplified from the *PRPH2* c.828+1G>A homozygous knockin hiPSC line (Figure 4B) and confirmed the predicted intron retention using dideoxy sequencing (Figures 4C and 4D). Our heterozygous and homozygous *PRPH2* c.828+1G>A PE-edited clones displayed reduced total (Figure 4E) and canonical (Figure 4F) *PRPH2* transcript levels with a concomitant increase in expression of the mis-spliced mutant variant (Figure 4G). Notably, the heterozygous clones exhibited an average of 4.7% mutant *PRPH2* transcript, whereas the homozygous clones showed 99.5%. Typically, mutant transcripts are degraded through nonsense-mediated decay (NMD).[39] To explore this, we inhibited NMD with cycloheximide (CHX) in the wild-type, heterozygous, and homozygous hiPSC lines (Figure S6). As a positive internal control, we evaluated the expression of SF2/ASF transcripts that increase upon CHX treatment (Figure S6A).[40] For both the wild-type and homozygous lines, we found no difference upon CHX treatment. In the heterozygous line, we found a decrease in canonical *PRPH2* transcript and an increase in mutant *PRPH2* transcript upon CHX treatment (Figures S6B and S6C). This suggests that the mutant transcript undergoes incomplete NMD.

Hernandez and colleagues previously reported that PRPH2 is localized not only in the outer segments of photoreceptors but also in hiPSCs. Notably, they suggested that hiPSCs predominantly express dimeric forms of PRPH2, whereas retinal organoids primarily express the monomeric form. In this study, we confirmed these findings by demonstrating that PRPH2 proteins are present in hiPSCs and retinal organoids (Figure S7).[41–43] Together, these findings strengthen previous work[25] and demonstrate that the *PRPH2* c.828+1G>A splice site mutation also leads to activation of the same cryptic splice site and causes intron retention. Further, it highlights the use of knockin hiPSCs for the screening of mis-splicing due to *PRPH2* mutations.

Toward *PRPH2* c.828+1G>A disease modeling and therapy

hiPSC-derived retinal organoids are three-dimensional structures that closely resemble the human fetal retina, effectively replicating its cell types and differentiation processes.[44,45] These organoids have proven valuable for modeling retinal diseases, as they can replicate the *in vivo* pathophysiology of the patient's condition *in vitro*.[45–49] *PRPH2*-mediated IRDs exhibit a wide variety of disease phenotypes, and the genotype-phenotype correlation remains unclear for many mutations, including the heterozygous *PRPH2* c.828+1G>A. Therefore, generating tools to facilitate model development for future studies is crucial. We aimed to test whether the edited heterozygous clone, which shares the same genotype as patients with the *PRPH2* c.828+1G>A variant (who are typically heterozygous for this mutation),[23] could successfully generate DD50/DD100 precursor retinal organoids. We also sought to determine if these organoids would express the mis-spliced transcript, which could enhance future disease modeling studies.

Retinal organoids derived from both wild-type and heterozygous *PRPH2* c.828+1G>A hiPSCs displayed normal morphology (Figure 5A) and exhibited OTX2-positive staining at differentiation day 50 (DD50) and DD100, indicating the presence of photoreceptor precursor cells. As expected, the abundance of apically localized OTX2-positive photoreceptor precursors increased between DD50 and DD100 (Figure 5B). This coincided with an increase in *PRPH2* transcript levels from the early stages of differentiation (DD50) to later stages (DD100) in the wild-type retinal organoids, correlating with the increased abundance and maturation of photoreceptor cells (Figure 5C). At DD50, there are no significant differences in total *PRPH2*, canonical *PRPH2*, or mutant *PRPH2* transcripts between wild-type and heterozygous retinal organoids. By DD100, total *PRPH2* transcript levels remain unchanged. However, in the *PRPH2* c.828+1G>A heterozygous retinal organoids, there is a significant decrease in canonical *PRPH2* transcript expression and a corresponding significant increase in the *PRPH2* mutant transcript levels (Figures 5D–5F).

NANOG and OCT3/4 in edited clones, indicating that both heterozygous and homozygous *PRPH2* c.828+1G>A knockin lines retain their pluripotent state following the prime editing event. Representative images are shown, with a scale bar representing 10 μm. (D) Immunofluorescence staining of PAX6 with Nestin, Brachyury with NCAM, and SOX17 with FOXA2 in edited clones after differentiation into ectodermal, mesodermal, and endodermal lineages, respectively. Representative images are shown, with a scale bar indicating 10 μm. Schematic created using BioRender.

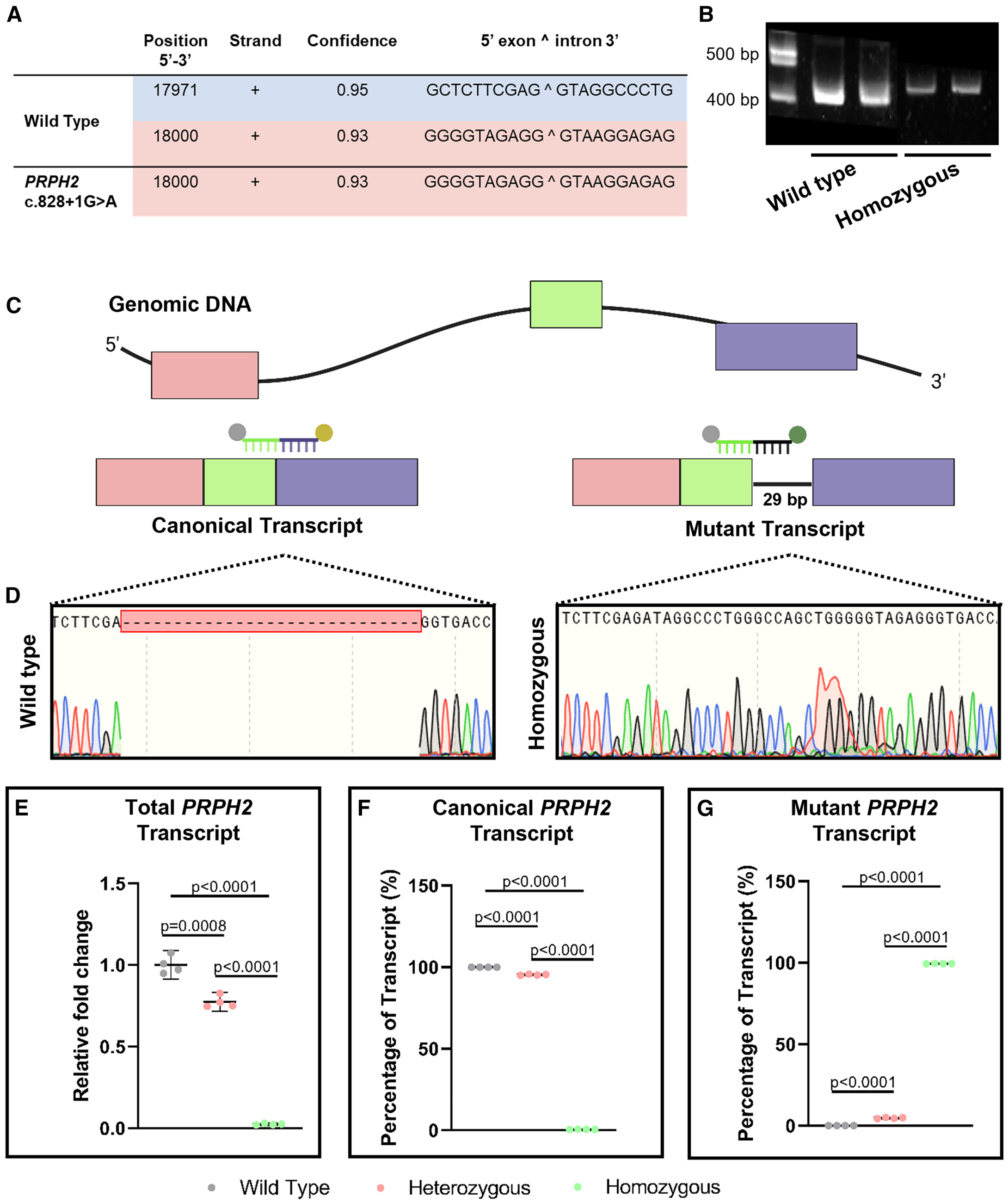

Figure 4. The *PRPH2* c.828 + 1G>A variant leads to activation of a cryptic splice site and intron retention

(A) NetGene2 analysis of the *PRPH2* c.828+1G>A splice site, with the canonical donor splice site marked in light blue and the cryptic splice site in light red. The canonical splice site is predicted to be lost. (B) Representative agarose gel showing PCR products using cDNA from wild-type and homozygous knockin hiPSC lines, demonstrating the predicted mutant transcript that includes 29 bp of intron 2. (C) Schematic depicting the predicted mis-spliced variant resulting from the *PRPH2* c.828+1G>A mutation, with highlighted positions of probes designed to specifically target the canonical (exon 2-exon 3 junction) and the mutant (exon 2-intron 2 junction) *PRPH2* transcripts. (D) Dideoxy sequencing traces of the PCR products shown in (B). (E and F) Quantification of *PRPH2* transcripts by real-time PCR. (E) Displays total *PRPH2* levels, (F) canonical *PRPH2* levels, and (G) mutant *PRPH2* levels. Data are expressed as mean ± 95% CI (n = 4). Schematic created using BioRender.

hiPSC-derived retinal organoids are also a promising preclinical model for evaluating the efficacy of therapeutic strategies. In this study, we aimed to develop tools to advance research in disease modeling and toward future therapeutic applications. Consequently, we set out to obtain proof-of-principle data and to evaluate PE as a potential method for correcting the *PRPH2*

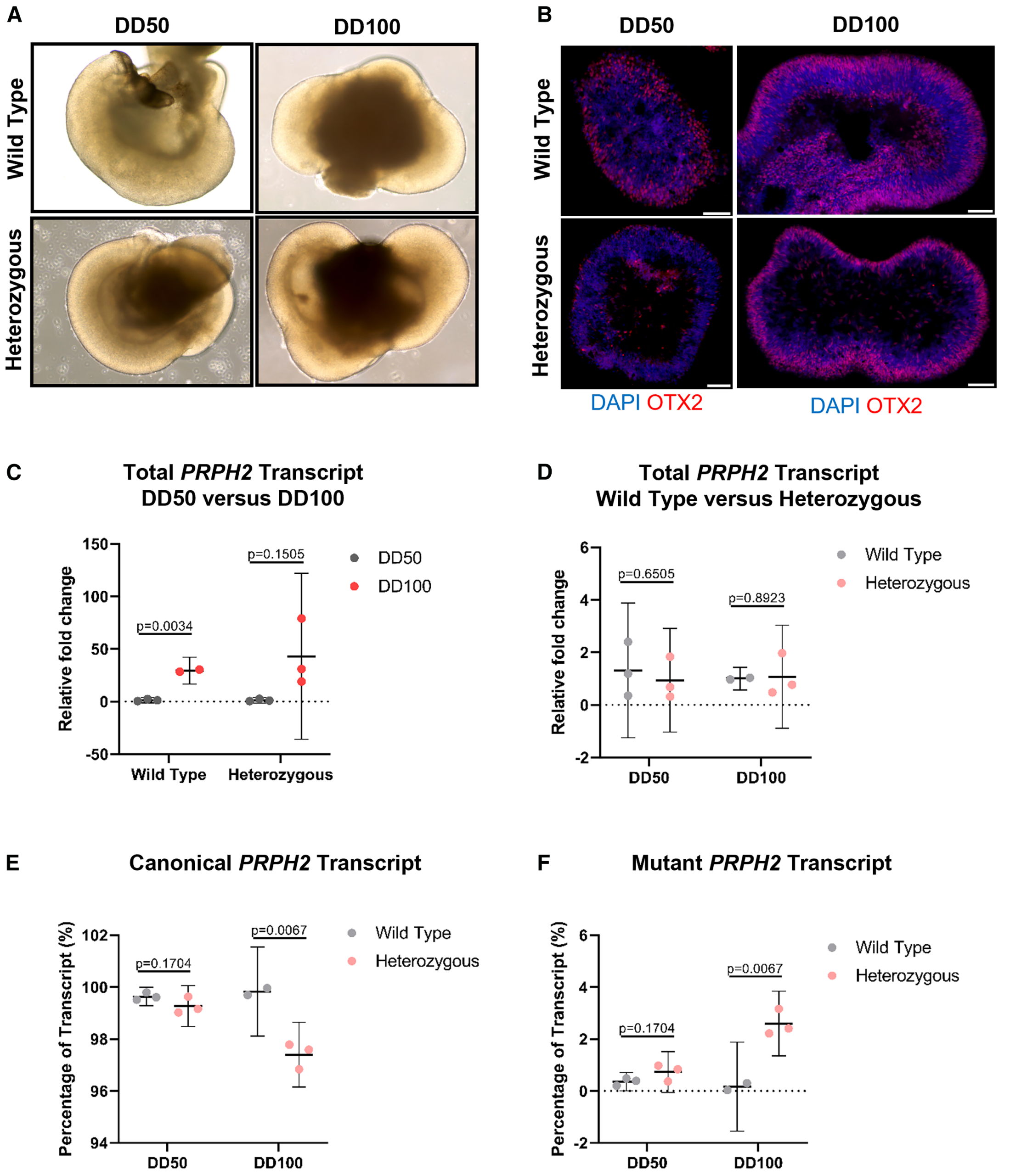

Figure 5. Successful generation of retinal organoids from wild-type and heterozygous *PRPH2* c.828+1G>A hiPSCs recapitulate aberrant splicing

(A) Bright-field images illustrate the normal morphology of wild-type and heterozygous retinal organoids at DD50 and DD100. (B) Immunofluorescence staining for OTX2 reveals the presence of photoreceptor precursor cells at DD50 and DD100 in both wild-type and heterozygous organoids. Representative images are shown, with a scale bar indicating 10 μm. (C–F) Quantification of *PRPH2* transcripts via real-time PCR. (C) Shows total *PRPH2* levels in wild-type and heterozygous retinal organoids, comparing expression at DD50 and DD100; (D) highlights total *PRPH2* expression at DD50 and DD100, comparing expression between wild-type and heterozygous organoids; (E) presents canonical *PRPH2* levels at DD50 and DD100, comparing expression between wild-type and heterozygous organoids; and (F) displays mutant *PRPH2* levels at DD50 and DD100, comparing expression between wild-type and heterozygous organoids. Data are expressed as mean ± 95% CI ($n \geq 2$).

c.828+1G>A variant. Using our previously established HEK293T homozygous *PRPH2* c.828+1G>A knockin line,[23] we screened the editing efficiency of 24 combinations of pegRNAs and nsgRNAs (Figures 6A and 6B; see Table S1 for sequences). pegRNA2 and nsgRNA3 demonstrated the highest editing efficiency (average of 18.27%) for correcting the *PRPH2* c.828+1G>A

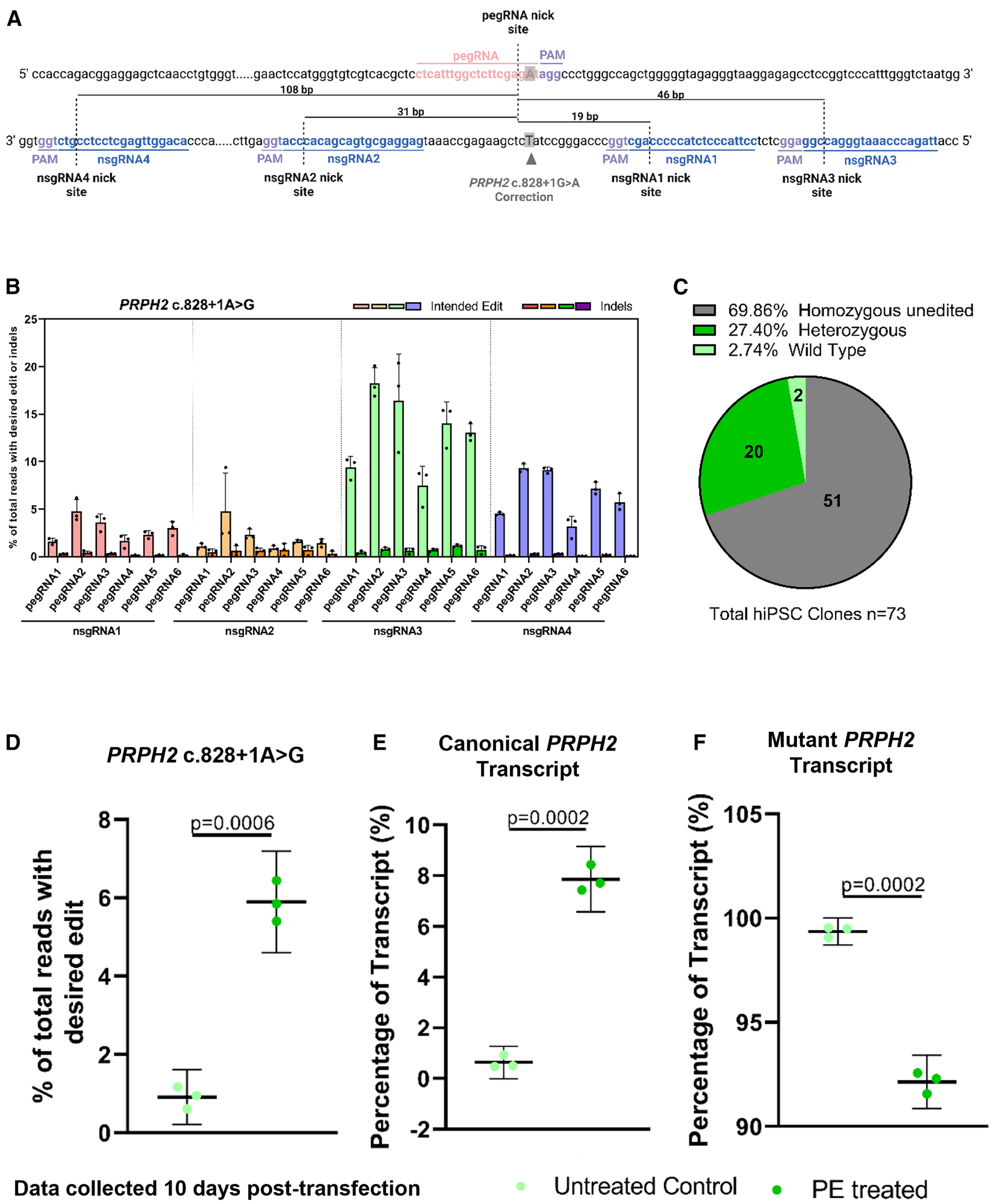

Figure 6. Prime editing corrects the *PRPH2* c.828 + 1G>A mutation in hiPSCs and restores canonical *PRPH2* transcript expression

(A) Schematic illustrating the prime editing designs tested for correcting the *PRPH2* c.828+1G>A splice mutant in hiPSCs. The diagram indicates the position of the edit, along with the pegRNA spacer, nsgRNA spacers, and the distance between the pegRNA and nsgRNA nick sites. (B) Editing efficiencies in HEK293T cells for all 24 combinations of pegRNA and nsgRNA tested to correct the *PRPH2* c.828+1G>A mutation. Data are presented as mean ± SD (n = 3). (C and D) Estimation of prime editing efficiency in correcting the *PRPH2* c.828+1G>A variant by (C) isolating *PRPH2* c.828+1G>A homozygous unedited, heterozygous, and wild-type clones through sorting of GFP-positive hiPSCs and (D) next-generation sequencing after 10 days of treatment (data expressed as mean ± 95% CI [n = 3]). (E and F) Quantification of *PRPH2* transcripts by real-time PCR. (E) presents canonical *PRPH2* levels and (F) shows mutant *PRPH2* levels. Data are expressed as mean ±95% CI (n = 3). Untreated control = c.828+1G>A homozygous line, PE treated = the treated c.828+1G>A homozygous line that shows wild-type genotype. Schematic created using BioRender.

variant, leading us to create an all-in-one construct with this specific combination (Figure 6B).

To further validate this tool for the correction of the *PRPH2* c.828+1G>A mutation, we used the previously generated homozygous hiPSC line. Isolation of GFP-positive clones revealed an overall editing efficiency of approximately 30%, comprising 2.7% wild-type and 27.4% heterozygous edited clones (Figure 6C). Correction analysis of pooled GFP-positive homozygous hiPSCs demonstrated an average genomic correction efficiency of 5.9% ten days post-transfection, as confirmed by NGS (Figures 6D, S8A, and S8B). While total *PRPH2* transcript levels remained unchanged (Figure S8C), we observed an average 7.86% reduction in the mutant transcript, accompanied by a proportional increase in the canonical transcript (Figures 6E and 6F).

These results demonstrate the feasibility of generating hiPSC-derived retinal organoids from the PE-edited *PRPH2* c.828+1G>A heterozygous hiPSC clone, positioning them as a valuable tool for future studies. Our findings provide proof-of-principle evidence that prime editing can correct the *PRPH2* c.828+1G>A variant at both the genomic and transcript levels. We observed a reduction in mutant transcript expression with an increase in canonical transcript expression, offering a quantifiable outcome measure for assessing therapeutic efficacy in future research.

DISCUSSION

PRPH2-mediated retinal degenerations represent a substantial burden for patients, ultimately resulting in progressive blindness. Despite the large number of mutations identified, there is still no clear genotype-phenotype correlation, and to date, there is no treatment available for *PRPH2*-mediated IRD patients. This highlights a critical need to develop tools and methods for understanding the pathobiology of disease and for the investigation of viable therapeutic interventions. In recent years, PE has demonstrated its ability to efficiently program precise genomic manipulations, making it a promising approach for single nucleotide variant disorders including *PRPH2*-mediated IRDs.[20–22] Our report models the *PRPH2* c.828+1G>A splice site variant in hiPSC and demonstrates that PE is an amenable approach for correcting this *PRPH2* variant in HEK293T cells and hiPSCs.

While PE shows promise for editing hiPSCs, further optimization of its design and delivery methods is needed to enhance efficiency.[50,51] Although mRNA-based delivery generally achieves higher survival and editing efficiency in hiPSC,[51] its synthesis can be expensive and labor-intensive. In this study, we present a method for delivering large PE plasmids to hiPSCs using Lipofectamine Stem reagent. To facilitate the screening of edited clones, we included a GFP reporter in our all-in-one plasmids. We initially hypothesized that cells with high GFP expression would be more likely to be edited. However, our results indicated that high GFP-expressing cells exhibited similar editing efficiency to low GFP-expressing cells (Figures 1C–1E). In our system, the prime editor and GFP are driven by different promoters, so GFP levels do not necessarily indicate editor expression, as they would with a 2A peptide system.[52] Therefore, the anticipated correlation between high GFP expression and editing likelihood does not hold true.

PRPH2-mediated diseases are typically inherited in an autosomal dominant manner,[53,54] meaning that a single damaging mutation in the *PRPH2* gene is sufficient to cause symptoms and disease. We previously reported on a 67-year-old patient with retinitis pigmentosa (RP) caused by a rare heterozygous *PRPH2* c.828+1G>A splice site mutation.[23] The rarity of some diseases presents a challenge for obtaining patient-derived material. The field of gene editing has helped to overcome these barriers by providing researchers with tools to introduce variants within otherwise healthy genetic backgrounds.[14,19] Using stem cell biology and the CRISPR PE tool, we generated heterozygous and homozygous hiPSC clones for the *PRPH2* c.828+1G>A variant. Further analysis of the edited clones confirmed the absence of off-target effects. The majority of predicted off-target sites exhibited three or more mismatches with the spacer sequence of pegRNA or nsgRNA, and no unintended modifications were detected, highlighting the precision of the PE approach in targeting the intended site. Additionally, no chromosomal abnormalities were detected in the edited lines, suggesting that the pegRNA and nsgRNA did not induce DSBs that could lead to chromosomal rearrangements. Furthermore, the edited lines retained their pluripotency and ability to differentiate into all three germ layers. Interestingly, our knockin hiPSC lines exhibited expression of a mis-spliced transcript, with heterozygous lines showing an average mutant transcript expression of 4.7% compared to 99.5% in homozygous lines (Figure 4). The aberrant splicing defect is due to the activation of a downstream cryptic splice site leading to the retention of 29 bp of intron 2 in the mutant transcript. This mutant transcript was previously detected in peripheral white blood cells collected from *PRPH2* patients with the prevalent c.828+3A>T variant.[25] Importantly, although the role of PRPH2 in hiPSCs is not fully understood, our results suggest that the expression of the mis-spliced transcript does not affect the pluripotency or differentiation capabilities of hiPSCs (Figure 3). The predicted protein product created by the mutant transcript from the *PRPH2* c.828+1G>A and c.828+3A>T variants would include an additional 10 amino acids (Figures S7A and S7B). These additional amino acids result in a premature stop codon at the beginning of exon 3, the last exon of *PRPH2*.[25] Similar to previous research, we demonstrated that hiPSCs predominantly express the dimeric form of PRPH2 protein, whereas retinal organoids primarily express the monomeric form.[41–43] The PRPH2 protein was detected in wild-type, c.828+1G>A heterozygous, and homozygous hiPSC lines (Figure S7C). To further determine whether the truncated mutant protein is expressed, alternative antibodies targeting specifically the mutant variant or mass spectrometry analysis would be required.

Following this, we generated DD50/DD100 precursor hiPSC-derived retinal organoids from our *PRPH2* c.828+1G>A heterozygous hiPSC line, alongside wild-type control organoids, to evaluate mis-splicing

in a tissue relevant context. The retinal organoids displayed normal morphology and expressed an increasing number of apically localized OTX2-positive photoreceptor precursor cells from DD50 to DD100.[55,56] At DD50, we detected nearly undetectable levels of *PRPH2* transcript in both wild-type and heterozygous organoids. By DD100, total *PRPH2* transcript levels significantly increased in the wild-type retinal organoids and showed a trend of increased expression in the c.828+1G>A heterozygous hiPSC-derived retinal organoids. Interestingly, unlike our findings in hiPSCs, there were no significant differences in total *PRPH2* transcript levels between wild-type and heterozygous organoids at these time points. This lack of difference may be attributed to the absence of maturated photoreceptors with fully formed segments in these earlier differentiation stages, resulting in lower total *PRPH2* levels. The PRPH2 protein is primarily localized to the disk membranes of the outer segments of cone and rod photoreceptors.[57] In hiPSC-derived retinal organoids, the outer segments begin to develop later in differentiation, specifically after DD150.[44,55] Notably, by DD100, we observed a significant decrease in canonical *PRPH2* transcript expression, while the levels of the mutant transcript significantly increased (Figures 5C–5F).

The mutant transcript produced by the *PRPH2* c.828+1G>A and c.828+3A>T variants may lead to either loss of function or a toxic gain of function.[25] We found upon CHX treatment in the c.828+1G>A heterozygous hiPSC line an increase in the mutant transcript. This suggests that to some degree this transcript goes under NMD. Additionally, we found no change in total mutant transcript upon treatment of the c.828+1G>A homozygous hiPSC line. Taken together, these data support the established rule that NMD is inefficient when premature termination codons occur in the last exon or within the final 50 nucleotides of the penultimate exon. In such cases, NMD is not expected to be triggered, allowing a mutant protein product to be produced.[58–60] However, as previously noted, detection of this mutant protein would require alternative strategies that enable to distinguish protein of similar molecular weights (Figures S7A and S7C). Importantly, it is believed that rod photoreceptors are more sensitive to the overall levels of PRPH2, while cone photoreceptors depend heavily on the proper functioning of PRPH2 complexes. Consequently, loss of function tends to primarily affect rods, whereas toxic gain of function has a more significant impact on cones.[5] As such, the mis-spliced transcript may play different roles in rod and cone photoreceptors. However, the genotype-phenotype correlation for the *PRPH2* c.828+1G>A variant remains unclear, and to our knowledge, there have been no studies using hiPSC-derived retinal organoids to investigate the disease phenotype associated with *PRPH2*-mediated IRDs. Our study paves the way for future research, not only to elucidate the retinal degenerative phenotypes caused by the *PRPH2* c.828 splice site variants using hiPSC retinal organoids but also to identify disease-specific morphological and molecular signatures that could apply to other *PRPH2*-mediated IRDs.

Lastly, we have demonstrated proof of principle that PE can correct the *PRPH2* c.828+1G>A mutation. Using the combination of pegRNA2 and nsgRNA3, we achieved 18.27% editing efficiency in HEK293T cells at 3 days post-transfection. In hiPSCs, 5.9% correction at the genomic level was found, which resulted in an average reduction of mutant transcript levels by 7.86%, while simultaneously increasing canonical transcript expression by the same proportion (Figures 6 and S8) at 10 days post-transfection. This finding provides molecular evidence that PE is a promising CRISPR gene editing tool for correcting the c.828+1G>A *PRPH2* mutation. Importantly, our research establishes a foundation for future studies evaluating the therapeutic efficacy of PE approaches in human post-mitotic cells, utilizing the *PRPH2* c.828+1G>A retinal organoid model.

In conclusion, we developed *PRPH2* c.828+1G>A PE-edited hiPSC lines that can serve as a platform for generating retinal organoids, which will be instrumental for future research on disease modeling and therapeutic development. Further, we strengthened previous findings on the c.828+3A>T *PRPH2* mutation, but in a retinal-tissue-specific manner, that mutations at the *PRPH2* c.828 splice site, including the c.828+1G>A, lead to activation of a cryptic splice site and cause intron retention. This highlights a potential common mode of retinal degeneration for this group of patients. Lastly, we demonstrated proof-of-principle data showing PE as a viable strategy to correct *PRPH2* variants that can be optimized in future work.

MATERIALS AND METHODS

Prime editing design and cloning strategy

The designs of pegRNA and nsgRNA for the *HEK3* and *FANCF* genes were sourced from a prior publication.[20] The pegRNA and nsgRNA sequences for the installation and correction of *PRPH2* c.828+1G>A were manually designed using SnapGene software. The pegRNA and nsgRNA sequences are summarized in Table S1.

A previously described modified version of the prime editor plasmid (pCMV-PE2, Addgene #132775), termed pCMV-PE(V4), was used.[61] HEK3, FANCF, and *PRPH2* c.828+1G>A knockin pegRNAs were cloned into pU6-pegRNA-GG-acceptor plasmid (Addgene #132777), and *PRPH2* c.828+1A>G correction pegRNAs and nsgRNAs were cloned into the pU6-tevopreq1-GG-acceptor plasmid (Addgene #174038) via Golden Gate Assembly, as previously reported.[22] HEK3, FANCF, and *PRPH2* c.828+1G>A knockin nsgRNAs were cloned into p7SK-spacer-acceptor plasmid (homemade) using the same strategy.

The all-in-one type 1 (T1) constructs were created following the assembly protocol outlined in Figure S1A. Similarly, all-in-one T2 to T8 constructs were generated by amplifying pegRNAs and nsgRNAs from acceptor plasmids using specific primers (listed in Table S2) to adjust their orientation within the plasmid. To summarize the process briefly: pCMV-PE(V4) was digested with Swa1 (New England Biolabs) overnight at 25°C. Fragments of pegRNAs were PCR-amplified from pegRNA acceptor plasmids such as pU6-pegRNA-HEK3, pU6-pegRNA-FANCF, pU6-pegRNA-*PRPH2*-c.828+1G>A-knockin, or pU6-pegRNA2-*PRPH2*-c.828+1G>A-correction. nsgRNA fragments were PCR-amplified from nsgRNA acceptor plasmids like

p7SK-nsgRNA-HEK3, p7SK-nsgRNA-FANCF, p7SK-nsgRNA-*PRPH2*-c.828+1G>A-knockin, or pU6-nsgRNA4-*PRPH2*-c.828+1 G>A-correction. Importantly, the T5 and T6 plasmids contain additional copies of pegRNA and nsgRNA, respectively. pegRNAs or nsgRNAs were amplified from the T1 plasmid using primers listed in Table S2. These sequences were then inserted into the T1 plasmid following digestion with the PCI1 restriction enzyme (New England Biolabs) overnight at 37°C. The final all-in-one constructs were cloned using In-Fusion technology, and the accuracy of the plasmid sequences was verified through dideoxy sequencing or Plasmid EZ (GENEWIZ services).

Cell culture and transfection

HEK293T cells were used for the validation of all-in-one constructs. HEK293T-*PRPH2*-c.828 + 1G>A-knockin cells, as previously established,[23] were utilized in our study to identify the optimal combination of pegRNA and nsgRNA for correcting the *PRPH2* c.828+1G>A mutation. HEK293T and HEK293T-*PRPH2*-c.828+1G>A-knockin cell lines were cultured in Dulbecco's modified Eagle medium (DMEM; Thermo Fisher Scientific) supplemented with 10% fetal bovine serum (FBS). To evaluate editing efficiency, all-in-one or separate systems were transfected into HEK293T or HEK293T-*PRPH2*-c.828+1G>A-knockin cells as previously described.[61] Cells were seeded at 50,000 cells/well in 24-well plates 24 h before transfection. For the transfection of the separate system, the plasmid constitution is 1050ng:393.75ng:78.75ng of pCMV-PE(V4), pU6-pegRNA, and p7SK/pU6-nsgRNA, respectively. For the transfection of all-in-one system, a total of 1,500 ng of DNA was delivered to the cells. Lipofectamine 2000 (Thermo Fisher Scientific) was mixed with the plasmid DNA at a 1:1 ratio. The cells were then collected after 72 h post-transfection for DNA extraction and analysis.

The work carried out in this manuscript was approved under Columbia University IRB protocols AAAF1849, AAAP0052, and under University of Pennsylvania IRB protocol 856063. Established hiPSCs were obtained from the Columbia Stem Cell Initiative at Columbia University Irving Medical Center (CUIMC) including CUIMCi002-A/FA0000011.[62] WTC-11 hiPSCs were purchased from the Coriell Institute. 1535-2 was reprogrammed using Invitrogen CytoTune Sendai virus from dermal fibroblasts of a healthy Caucasian male in his mid-40s. hiPSC colonies were cultured in mTeSR Plus medium (StemCell Technologies) on Matrigel-coated dishes (Corning). hiPSCs were dissociated using ReLeSR (StemCell Technologies) and seeded at 150,000–200,000 per well in 12-well Matrigel-coated plates 24 h prior to transfection. Transfection complexes consisted of 1.5 μg, 2.25 μg, or 3 μg of DNA mixed with 4 μL, 5 μL, or 6 μL of Lipofectamine Stem (Thermo Fisher Scientific). Seventy-two hours post-transfection, GFP-positive cells were sorted using FACS into either pooled or single cells in 96-well Matrigel-coated plates containing mTeSR Plus medium supplemented with CloneR2 (StemCell Technologies). Pooled-sorted hiPSCs were grown until reaching 95%–100% confluency, at which point DNA was extracted to confirm editing via dideoxy sequencing and/or Amplicon EZ (GENEWIZ services). Single cells were expanded, and a portion of cells from each clone was harvested for genotyping analysis using dideoxy sequencing. Positive clones were subsequently expanded further and subjected to confirmatory sequencing using both dideoxy sequencing and Amplicon EZ to validate the desired genotypes.

Fluorescence-activated cell sorting

Transfected hiPSCs were dissociated using TrypLE Select Enzyme (Thermo Fisher Scientific), suspended in Dulbecco's phosphate-buffered saline (DPBS; Thermo Fisher Scientific) and filtered through a 35 μm cell strainer (Thermo Fisher Scientific) to obtain a single cell suspension. hiPSCs were sorted using a BD Influx System and BD FACS Software. In the experiment assessing the correlation between GFP expression intensity and editing efficiency, high-expressing GFP and low-expressing GFP populations were sorted based on the criteria outlined in Figure 1C. For all subsequent experiments, GFP-positive cells, encompassing both high and low levels of GFP expression, were sorted.

Retinal organoids differentiation

Retinal organoid differentiation from hiPSCs followed a previously described protocol.[63] Initially, hiPSCs cultured on Matrigel were dissociated using ReLeSR and seeded into microwells of an agarose micro-mold (agarose microwell array seeding and scraping—AMASS method) in mTeSR supplemented with blebbistatin to induce embryoid body (EB) formation. Over the first 3 days (days 0–3), the medium was gradually transitioned from mTeSR Plus to neural induction medium 1 (NIM1), with one-third medium exchanges on days 1 and 2. By day 7, EBs were plated onto Matrigel-coated wells to initiate neuroepithelium differentiation, with a subsequent switch to neural induction medium 2 (NIM2) by day 16. By day 28, we performed checkerboard scraping and lifting of the neuroepithelia using a cell scraper. From this point onward, retinal organoids were maintained under low attachment conditions using Poly-HEMA (Poly 2-hydroxyethyl methacrylate)-coated plates. At day 42, retinal lamination medium 1 (RLM1) was introduced to promote further maturation, followed by RLM2 at day 70 and RLM3 at day 98, and varied concentrations of retinoic acid. Organoids were collected at DD50 and DD100 for downstream experiments.

In silico analysis of *PRPH2* c.828+1G>A variant

The impact of the *PRPH2* c.828+1G>A mutation on the donor splice site of exon 2 was assessed using the Netgene2 program (http://www.cbs.dtu.dk/services/NetGene2), as previously described.[25,64]

Prime editing efficiency analysis

Cells from each well were washed with DPBS and then resuspended in 30 μL of DPBS. Subsequently, DNA extraction was carried out following a previously reported method.[23] To assess editing efficiency, we either isolated multiple clones derived from single GFP-positive cells and determined the editing rate by dideoxy sequencing, calculating the percentage based on the total number of clones screened, or we amplified the target locus using primers with Illumina adapters and barcode sequences for Amplicon EZ. Detailed

primer sequences are provided in Table S3. Dideoxy sequencing results were obtained from the manufacturer's platform and subsequently aligned and analyzed; in some cases, ICE analysis was also conducted. Amplicon EZ results were downloaded and demultiplexed into individual files for analysis using CRISPResso2. The demultiplexing method involved the use of barcoded pairs of primers (forward and reverse). Unique pairs were selected to amplify the targeted genomic loci and then pooled together for processing/ sequencing. R1 and R2 files from Amplicon EZ sequencing were aligned and merged into single-end reads using BBMerge from the BBTools suite. Resulting FASTQ files were then segregated by their unique forward and reverse barcodes in python using SeqIO package tools from the Bio library and Bio.Seq tools from the Seq library. Sequestered subsets were then fed into CRISPResso2 and analyzed for editing efficiencies.

Off-target prediction and analysis

In this study, we conducted targeted off-target analysis as previously reported.[19] Off-target predictions were carried out using the online tool Cas-OFFinder.[65] All predicted off-targets with three or fewer mismatches that could be specifically amplified by PCR were thoroughly examined. This study focused on genomic regions that could be targeted by pegRNAs and nsgRNAs. Selected off-targets were amplified using PCR (with primers detailed in Table S3), and the resulting products were then sequenced using dideoxy sequencing.

RNA extraction and quantitative real-time PCR

Total RNA from hiPSCs and hiPSC-derived retinal organoids was extracted using the RNeasy Extraction Kit (Qiagen), according to the manufacturer's instructions. Three to five hiPSC-derived retinal organoids were pooled together for RNA extraction. DNase I treatment (Invitrogen) was performed to prevent genomic DNA contamination. The reverse transcription reaction utilized the Superscript III reverse transcription kit, with an oligo(dT)20 (Invitrogen) for cDNA synthesis.

Total, canonical, and mutant *PRPH2* transcripts were quantitatively detected using specific TaqMan probes (refer to Table S4 for primers and probes sequences). Real-time PCR was carried out using the PrimeTime Gene Expression Master Mix (Integrated DNA Technologies) and the CFX Connect real-time PCR detection system (Bio-Rad) to quantify the gene expression levels. Endogenous *β-actin* mRNA expression levels were determined using the PrimeTime Std qPCR assay (Hs.PT.39a.22214847). Each well was assigned either probes for total *PRPH2* transcripts along with *β-actin* or probes for canonical and mutant *PRPH2* transcripts. The Ct values were averaged for each in-plate technical duplicate. For the quantification of total *PRPH2* transcript, the averaged Ct was normalized as the difference in Ct value (ΔCt) between the total *PRPH2* transcript and *β-actin*. The variation was reported as a relative fold change [2(-ΔΔCt)] as previously reported.[66] The variation of canonical and mutant *PRPH2* transcripts was reported as the percentage of transcripts. The results are presented as mean ± 95% confidence interval (CI). CHX was dissolved in DMSO and subsequently diluted in mTeSR Plus medium to a final concentration of 100 μg/mL. Wild-type, heterozygous, and homozygous hiPSCs were treated with CHX at 37°C for 6 h. Control cells were treated with an equivalent volume of DMSO. Following treatment, cells were washed thoroughly with PBS, and total RNA was extracted for downstream analyses.

Immunofluorescence staining

For pluripotency analysis and germ layer potential, hiPSC clones were seeded on Matrigel-coated chamber slides (CELLTREAT Scientific Products). For pluripotency analysis, cells were fixed once colonies formed, and for germ layer potential, cells were cultured using the STEMdiff Trilineage Differentiation Kit (Stem Cell Technologies) according to the manufacturer's protocol and then fixed. Cells were fixed in 4% paraformaldehyde in DPBS for 30 min at room temperature. Retinal organoids were fixed as above and then briefly washed in DPBS followed by cryoprotection in 15% and then 30% sucrose in DPBS and subsequently embedded in Tissue-Tek O.C. T. Compound (Sakura, Finetek), frozen and stored at −20°C. Subsequently, 8 μM cryosections were made with a Leica CM3050S cryostat (Leica Microsystems) and placed on slides that were stored at −20°C for future use.

For analysis by immunohistochemistry, the slides containing cells or retinal organoid sections were blocked and permeabilized in 10% normal goat serum, 1% Triton X-100, and 1% bovine serum albumin (BSA) in DPBS for 1 h at room temperature. Slides were incubated with primary antibodies diluted in 10% normal goat serum, 1% Triton X-100, and 1% BSA in DPBS at 4°C overnight. The next day, slides were washed in DPBS three times and incubated with secondary antibody in 1% BSA in DPBS for 1 h at room temperature. Subsequently, slides were washed three times in DPBS and mounted using VECTASHIELD Vibrance Antifade Mounting Medium with DAPI (H-1800).

The following primary antibodies were used for pluripotency analysis: OCT3/4 (1:200; R&D Systems) and NANOG (1:200; R&D Systems). The following primary antibodies were used for germ layer confirmation: ectoderm – PAX6 (1:200; Invitrogen) and Nestin (1:200; Sigma-Aldrich), mesoderm – Brachyury (1:200; R&D Systems) and NCAM (1:200; STEMCELL Technologies), and endoderm – SOX17 (1:200; R&D Systems) and FOXA2 (1:200; R&D Systems). The following primary antibodies were used on retinal organoids: OTX2 (1:250; R&D Systems). The following secondary antibodies were used: Donkey anti-Goat conjugated to Alexa 488 (1:500, A-11055, Invitrogen), Donkey anti-Rabbit conjugated to Alexa 555 (1:500, A-31572, Invitrogen), Goat anti-Mouse conjugated to Alexa 488 (1:500, A-32723, Invitrogen), and Goat anti-Mouse conjugated to Alexa 555 (1:500, A-21425, Invitrogen). Immunofluorescence visualization and imaging were performed using a Nikon Eclipse 80i upright fluorescent microscope.

Protein isolation and immunoblots

hiPSCs and wild-type retinal organoids (DD225) were collected and directly lysed in 200 μL of 1× SDS Laemmli Buffer (Bio-Rad)

supplemented with Bond-Breaker TCEP solution (Thermo Fisher Scientific). Samples were then sonicated for five cycles at 25% power (3 s on/off per cycle) and incubated at 95°C for 5 min. The resulting lysates were loaded onto gels.

Proteins were separated on 4%–15% Mini-PROTEAN TGX gels (Bio-Rad) and transferred onto PVDF membranes (Thermo Fisher Scientific). Membranes were blocked for 30 min in EveryBlot blocking buffer and incubated overnight at 4°C with the primary antibody diluted in the same buffer. A rabbit polyclonal anti-PRPH2 antibody (1:1000; ProteinTech, 18109-1-AP) was used.

Following incubation, the membranes were washed three times with Tris-buffered saline (TBS) containing 0.1% Tween 20 (TBS-T 0.1%) for 8 min each. They were then incubated for 2 h at room temperature with a donkey anti-rabbit IgG-HRP secondary antibody (1:2,000; NA934V). After three additional washes with TBS-T 0.1%, protein bands were detected using the iBright 1500 Imaging System and SuperSignal West Pico PLUS Chemiluminescent Substrate (Thermo Fisher Scientific).

The membranes were then stripped with Restore PLUS Western Blot Stripping Buffer (15 min), washed with TBS-T 0.1%, and re-blocked for 30 min in EveryBlot Blocking Buffer. They were subsequently incubated overnight at 4°C with a mouse monoclonal anti-β-actin HRP-conjugated antibody (1:1,000; Cell Signaling, 12262S). The following day, the membranes were washed again three times with TBS-T 0.1% for 8 min each, and bands were visualized using the same protocol described above.

Statistical analysis

Statistical analyses were conducted using GraphPad Prism software v.9.3.1. For comparisons involving three groups, we initially performed Welch's ANOVA, followed by an unpaired Student's t test with Welch's correction if significant differences were detected. For comparisons between two groups, we applied the unpaired Student's t test with Welch's correction. Quantitative data are presented as mean ± 95% CI or standard deviation (SD), as specified in each figure legend.

DATA AND CODE AVAILABILITY

The original data presented in this study are included in the article/additional files, and further inquiries can be directed to the corresponding author.

ACKNOWLEDGMENTS

We would like to thank members of the PrimeSight Lab and the Jonas Children's Vision Care (JCVC) laboratory for their support and comradery. In particular, we would like to thank Wen-Hsuan Wu and Jimmy Duong for statistical analysis support. Further, we would like to thank the lab of David R. Liu for helping with implementing our next-generation sequencing workflow and prime editing advice. This work was supported by a private donation to the JCVC team and in part by a Foundation Fighting Blindness Individual Investigator Research Award TA-GT-0623-0859-UPA, the Curing Retinal Blindness Foundation (CRBF), and National Institutes of Health (NIH) grant R01EY034952, all to P.M.J.Q. Additionally, P.M.J.Q. and the PrimeSight Lab are supported by Startup funds from the Department of Ophthalmology at the University of Pennsylvania, the F.M. Kirby Foundation, the Paul and Evanina Bell Mackall Foundation Trust, Research to Prevent Blindness, the Costa Family Fund, Iris Bennett Siegel Retinitis Pigmentosa Research Fund, and NIH grant P30EY001583. B.L.d.C. is a recipient of the Capes PhD scholarship. K.M.H. is a recipient of the National Institutes of Health Ruth L. Kirschstein Predoctoral Individual National Research Service Award (F31 NS137770). C.D.M. is supported by the National Institutes of Health (DP2 MH132944). JCVC laboratory is supported by the National Institutes of Health (U01 EY030580, U54OD020351, R24EY028758, R24EY027285, 5P30EY019007, R01EY018213, R01EY024698, and R01EY033770), NYEE Foundation, the Foundation Fighting Blindness TA-GT-0321-0802-COLU-TRAP, Lynette & Richard Jaffe Foundation, Nancy & Kobi Karp, the Crowley Family Fund, the Rosenbaum Family Foundation, the Gebroe Family Foundation, the Research to Prevent Blindness (RPB) Physician-Scientist Award, and unrestricted funds from RPB, New York, NY, U.S.A. Flow cytometry experiments used the resources of the Columbia Center for Translational Immunology (CCTI) Flow Cytometry Core, supported in part by the Office of the Director, National Institutes of Health under award S10OD020056. The funding organizations had no role in the design or conduct of this research. All schematic images were generated using BioRender.

AUTHOR CONTRIBUTIONS

Conceptualization, B.L.d.C., Y.-T.T., and P.M.J.Q.; formal analysis: B.L.d.C. and P.M.J.Q.; funding acquisition, S.H.T. and P.M.J.Q.; investigation, B.L.d.C., K.M.H., K.T., S.M.C., S.L., J.R.L.d.C., N.D.N., C.D.M., S.H.T., and P.M.J.Q.; methodology, B.L.d.C., K.M.H., K.T., Y.-T.T., S.M.C., and P.M.J.Q.; resources, C.D.M. and S.H.T.; software, S.M.C.; supervision, C.D.M., S.H.T., and P.M.J.Q.; validation, B.L.d.C. and P.M.J.Q; visualization, B.L.d.C., K.M.H., K.T., and P.M.J.Q.; writing – original draft, B.L.d.C. and P.M.J.Q.; writing – review & editing, all authors.

DECLARATION OF INTERESTS

S.H.T. receives financial support from Emendo and is on the scientific and clinical advisory board for Medical Excellence Capital and Nanoscope Therapeutics. Columbia University has filed a patent application (WO 2023/220732 A1) related to PRPH2 for which B.L.d.C., Y.-T.T., S.M.C., S.H.T., and P.M.J.Q. are listed as inventors.

DECLARATION OF GENERATIVE AI AND AI-ASSISTED TECHNOLOGIES IN THE WRITING PROCESS

During the preparation of this work, the author(s) used ChatGPT in order to improve the readability of the manuscript. After using this tool/service, the author(s) reviewed and edited the content as needed and take(s) full responsibility for the content of the publication.

SUPPLEMENTAL INFORMATION

Supplemental information can be found online at https://doi.org/10.1016/j.omtn.2025.102740.

REFERENCES

1. Boon, C.J.F., den Hollander, A.I., Hoyng, C.B., Cremers, F.P.M., Klevering, B.J., and Keunen, J.E.E. (2008). The Spectrum of Retinal Dystrophies Caused by Mutations in the Peripherin/RDS Gene. Prog. Retin. Eye Res. *27*, 213–235. https://doi.org/10.1016/j.preteyeres.2008.01.002.

2. Peeters, M.H.C.A., Khan, M., Rooijakkers, A.A.M.B., Mulders, T., Haer-Wigman, L., Boon, C.J.F., Klaver, C.C.W., van den Born, L.I., Hoyng, C.B., Cremers, F.P.M., et al. (2021). PRPH2 mutation update: In silico assessment of 245 reported and 7 novel variants in patients with retinal disease. Hum. Mutat. *42*, 1521–1547. https://doi.org/10.1002/humu.24275.

3. Stone, E.M., Andorf, J.L., Whitmore, S.S., DeLuca, A.P., Giacalone, J.C., Streb, L.M., Braun, T.A., Mullins, R.F., Scheetz, T.E., Sheffield, V.C., and Tucker, B.A. (2017). Clinically Focused Molecular Investigation of 1000 Consecutive Families with Inherited Retinal Disease. Ophthalmology *124*, 1314–1331. https://doi.org/10.1016/j.ophtha.2017.04.008.

4. Dias, M.F., Joo, K., Kemp, J.A., Fialho, S.L., da Silva Cunha, A., Woo, S.J., and Kwon, Y.J. (2018). Molecular genetics and emerging therapies for retinitis pigmentosa: Basic research and clinical perspectives. Prog. Retin. Eye Res. *63*, 107–131. https://doi.org/10.1016/j.preteyeres.2017.10.004.

5. Conley, S.M., and Naash, M.I. (2014). Gene therapy for PRPH2-Associated ocular disease: Challenges and prospects. Cold Spring Harb. Perspect. Med. *4*, a017376. https://doi.org/10.1101/cshperspect.a017376.

6. Ali, R.R., Sarra, G.-M., Stephens, C., De Alwis, M., Bainbridge, J.W.B., Munro, P.M., Fauser, S., Reichel1, M.B., Kinnon, C., Hunt, D.M., et al. (2000). Restoration of photoreceptor ultrastructure and function in retinal degeneration slow mice by gene therapy. Nat. Genet. *25*, 306–310. https://doi.org/10.1038/77068.

7. Sarra, G.-M., Stephens, C., De Alwis, M., Bainbridge, J.W.B., Smith, A.J., Thrasher, A.J., and Ali, R.R. (2001). Gene replacement therapy in the retinal degeneration slow (rds) mouse: the effect on retinal degeneration following partial transduction of the retina. Hum. Mol. Genet. *10*, 2353–2361. https://doi.org/10.1093/hmg/10.21.2353.

8. Cai, X., Nash, Z., Conley, S.M., Fliesler, S.J., Cooper, M.J., and Naash, M.I. (2009). A partial structural and functional rescue of a retinitis pigmentosa model with compacted DNA nanoparticles. PLoS One *4*, e5290. https://doi.org/10.1371/journal.pone.0005290.

9. Cai, X., Conley, S.M., Nash, Z., Fliesler, S.J., Cooper, M.J., and Naash, M.I. (2010). Gene delivery to mitotic and postmitotic photoreceptors Via compacted DNA nanoparticles results in improved phenotype in a mouse model of retinitis pigmentosa. FASEB J. *24*, 1178–1191. https://doi.org/10.1096/fj.09-139147.

10. Schlichtenbrede, F.C., da Cruz, L., Stephens, C., Smith, A.J., Georgiadis, A., Thrasher, A.J., Bainbridge, J.W.B., Seeliger, M.W., and Ali, R.R. (2003). Long-term evaluation of retinal function in Prph2Rd2/Rd2 mice following AAV-mediated gene replacement therapy. J. Gene Med. *5*, 757–764. https://doi.org/10.1002/jgm.401.

11. Farjo, R., Skaggs, J.S., Nagel, B.A., Quiambao, A.B., Nash, Z.A., Fliesler, S.J., and Naash, M.I. (2006). Retention of function without normal disc morphogenesis occurs in cone but not rod photoreceptors. J. Cell Biol. *173*, 59–68. https://doi.org/10.1083/jcb.200509036.

12. Ding, X.Q., Nour, M., Ritter, L.M., Goldberg, A.F.X., Fliesler, S.J., and Naash, M.I. (2004). The R172W mutation in peripherin/rds causes a cone-rod dystophy in transgenic mice. Hum. Mol. Genet. *13*, 2075–2087. https://doi.org/10.1093/hmg/ddh211.

13. Conley, S.M., Stuck, M.W., Burnett, J.L., Chakraborty, D., Azadi, S., Fliesler, S.J., and Naash, M.I. (2014). Insights into the mechanisms of macular degeneration associated with the R172W mutation in RDS. Hum. Mol. Genet. *23*, 3102–3114. https://doi.org/10.1093/hmg/ddu014.

14. Cerna-Chavez, R., Ortega-Gasco, A., Baig, H.M.A., Ehrenreich, N., Metais, T., Scandura, M.J., Bujakowska, K., Pierce, E.A., and Garita-Hernandez, M. (2024). Optimized Prime Editing of Human Induced Pluripotent Stem Cells to Efficiently Generate Isogenic Models of Mendelian Diseases. Int. J. Mol. Sci. *26*, 114. https://doi.org/10.3390/ijms26010114.

15. Volpato, V., and Webber, C. (2020). Addressing Variability in iPSC-Derived Models of Human Disease: Guidelines to Promote Reproducibility. Dis. Model Mech. *13*, dmm042317. https://doi.org/10.1242/dmm.042317.

16. Liang, G., and Zhang, Y. (2013). Embryonic stem cell and induced pluripotent stem cell: An epigenetic perspective. Cell Res. *23*, 49–69. https://doi.org/10.1038/cr.2012.175.

17. Nandy, K., Babu, D., Rani, S., Joshi, G., Ijee, S., George, A., Palani, D., Premkumar, C., Rajesh, P., Vijayanand, S., et al. (2023). Efficient gene editing in induced pluripotent stem cells enabled by an inducible adenine base editor with tunable expression. Sci. Rep. *13*, 21953. https://doi.org/10.1038/s41598-023-42174-2.

18. Pierce, E.A., Aleman, T.S., Jayasundera, K.T., Ashimatey, B.S., Kim, K., Rashid, A., Jaskolka, M.C., Myers, R.L., Lam, B.L., Bailey, S.T., et al. (2024). Gene Editing for CEP290 -Associated Retinal Degeneration. N. Engl. J. Med. *390*, 1972–1984. https://doi.org/10.1056/nejmoa2309915.

19. Siles, L., Ruiz-Nogales, S., Navinés-Ferrer, A., Méndez-Vendrell, P., and Pomares, E. (2023). Efficient correction of ABCA4 variants by CRISPR-Cas9 in hiPSCs derived from Stargardt disease patients. Mol. Ther. Nucleic Acids *32*, 64–79. https://doi.org/10.1016/j.omtn.2023.02.032.

20. Anzalone, A.V., Randolph, P.B., Davis, J.R., Sousa, A.A., Koblan, L.W., Levy, J.M., Chen, P.J., Wilson, C., Newby, G.A., Raguram, A., and Liu, D.R. (2019). Search-and-replace genome editing without double-strand breaks or donor DNA. Nature *576*, 149–157. https://doi.org/10.1038/s41586-019-1711-4.

21. Costa, B.L.d., Levi, S.R., Eulau, E., Tsai, Y.-T., and Quinn, P.M.J. (2021). Prime Editing for Inherited Retinal Diseases. Front. Genome Ed. *3*, 775330. https://doi.org/10.3389/fgeed.2021.775330.

22. Tsai, Y.-T., da Costa, B.L., Nolan, N.D., Caruso, S.M., Jenny, L.A., Levi, S.R., Tsang, S.H., and Quinn, P.M.J. (2023). Prime Editing for the Installation and Correction of Mutations Causing Inherited Retinal Disease: A Brief Methodology. Methods Mol. Biol. *2560*, 313–331. https://doi.org/10.1007/978-1-0716-2651-1_29.

23. Caruso, S.M., Tsai, Y.-T., da Costa, B.L., Kolesnikova, M., Jenny, L.A., Tsang, S.H., and Quinn, P.M.J. (2023). Prime Editing Strategy to Install the PRPH2 c.828+1G>A Mutation. Adv. Exp. Med. Biol. *1415*, 97–102. https://doi.org/10.1007/978-3-031-27681-1_15.

24. Shankar, S.P., Hughbanks-Wheaton, D.K., Birch, D.G., Sullivan, L.S., Conneely, K. N., Bowne, S.J., Stone, E.M., and Daiger, S.P. (2016). Autosomal dominant retinal dystrophies caused by a founder splice site mutation, c.828+3A>T, in PRPH2 and protein haplotypes in trans as modifiers. Investig. Ophthalmol. Vis. Sci. *57*, 349–359. https://doi.org/10.1167/iovs.15-16965.

25. Shankar, S.P., Birch, D.G., Ruiz, R.S., Hughbanks-Wheaton, D.K., Sullivan, L.S., Bowne, S.J., Stone, E.M., and Daiger, S.P. (2015). Founder effect of a c.828+3A>T splice site mutation in peripherin 2 (PRPH2) causing autosomal dominant retinal dystrophies. JAMA Ophthalmol. *133*, 511–517. https://doi.org/10.1001/jamaophthalmol.2014.6115.

26. Gamundi, M.J., Hernan, I., Muntanyola, M., Trujillo, M.J., García-Sandoval, B., Ayuso, C., Baiget, M., and Carballo, M. (2007). High prevalence of mutations in peripherin/RDS in autosomal dominant macular dystrophies in a Spanish population. Mol. Vis. *13*, 1031–1037.

27. Telmer, C.A., Retchless, A.C., Kinsey, A.D., Conley, Y., Rigatti, B., Gorin, M.B., and Jarvik, J.W. (2003). Detection and assignment of mutations and minihaplotypes in human DNA using peptide mass signature genotyping (PMSG): Application to the human RDS/peripherin gene. Genome Res. *13*, 1944–1951. https://doi.org/10.1101/gr.995103.

28. Zampaglione, E., Kinde, B., Place, E.M., Navarro-Gomez, D., Maher, M., Jamshidi, F., Nassiri, S., Mazzone, J.A., Finn, C., Schlegel, D., et al. (2020). Copy-number variation contributes 9% of pathogenicity in the inherited retinal degenerations. Genet. Med. *22*, 1079–1087. https://doi.org/10.1038/s41436-020-0759-8.

29. Palmer, A.C., Egan, J.B., and Shearwin, K.E. (2011). Transcriptional interference by RNA polymerase pausing and dislodgement of transcription factors. Transcription *2*, 9–14. https://doi.org/10.4161/trns.2.1.13511.

30. Simons, R.W., Hoopes, B.C., McClure, W.R., and Kleckner, N. (1983). Three promoters near the termini of IS10: pIN, pOUT, and pIII. Cell *34*, 673–682. https://doi.org/10.1016/0092-8674(83)90400-2.

31. Nigumann, P., Redik, K., Mätlik, K., and Speek, M. (2002). Many human genes are transcribed from the antisense promoter of L1 retrotransposon. Genomics *79*, 628–634. https://doi.org/10.1006/geno.2002.6758.

32. Callen, B.P., Shearwin, K.E., and Egan, J.B. (2004). Transcriptional interference between convergent promoters caused by elongation over the promoter. Mol. Cell *14*, 647–656. https://doi.org/10.1016/j.molcel.2004.05.010.

33. Carquet, M., Pompon, D., and Truan, G. (2015). Transcription interference and ORF nature strongly affect promoter strength in a reconstituted metabolic pathway. Front. Bioeng. Biotechnol. *3*, 21. https://doi.org/10.3389/fbioe.2015.00021.

34. Søndergaard, J.N., Geng, K., Sommerauer, C., Atanasoai, I., Yin, X., and Kutter, C. (2020). Successful delivery of large-size CRISPR/Cas9 vectors in hard-to-transfect human cells using small plasmids. Commun. Biol. *3*, 319. https://doi.org/10.1038/s42003-020-1045-7.

35. Papathanasiou, S., Markoulaki, S., Blaine, L.J., Leibowitz, M.L., Zhang, C.Z., Jaenisch, R., and Pellman, D. (2021). Whole chromosome loss and genomic instability in mouse embryos after CRISPR-Cas9 genome editing. Nat. Commun. *12*, 5855. https://doi.org/10.1038/s41467-021-26097-y.

36. Adikusuma, F., Piltz, S., Corbett, M.A., Turvey, M., McColl, S.R., Helbig, K.J., Beard, M.R., Hughes, J., Pomerantz, R.T., and Thomas, P.Q. (2018). Large deletions induced by Cas9 cleavage. Nature *560*, E8–E9. https://doi.org/10.1038/s41586-018-0380-z.

37. Weckselblatt, B., and Rudd, M.K. (2015). Human Structural Variation: Mechanisms of Chromosome Rearrangements. Trends Genet. *31*, 587–599. https://doi.org/10.1016/j.tig.2015.05.010.

38. Fiumara, M., Ferrari, S., Omer-Javed, A., Beretta, S., Albano, L., Canarutto, D., Varesi, A., Gaddoni, C., Brombin, C., Cugnata, F., et al. (2024). Genotoxic effects of base and prime editing in human hematopoietic stem cells. Nat. Biotechnol. *42*, 877–891. https://doi.org/10.1038/s41587-023-01915-4.

39. Wilkinson, M.F. (2005). A new function for nonsense-mediated mRNA-decay factors. Trends Genet. *21*, 143–148. https://doi.org/10.1016/j.tig.2005.01.007.

40. Sun, S., Zhang, Z., Sinha, R., Karni, R., and Krainer, A.R. (2010). SF2/ASF autoregulation involves multiple layers of post-transcriptional and translational control. Nat. Struct. Mol. Biol. *17*, 306–312. https://doi.org/10.1038/nsmb.1750.

41. Hernandez, J., Huffman, K., and Borooah, S. (2024). Peripherin-2 expression: beyond the outer segment. Investig. Ophthalmol. Vis. Sci. *65*, 1299.

42. Strayve, D., Makia, M.S., Kakakhel, M., Sakthivel, H., Conley, S.M., Al-Ubaidi, M.R., and Naash, M.I. (2020). ROM1 contributes to phenotypic heterogeneity in PRPH2-associated retinal disease. Hum. Mol. Genet. *29*, 2708–2722. https://doi.org/10.1093/hmg/ddaa160.

43. Zulliger, R., Conley, S.M., Mwoyosvi, M.L., Al-Ubaidi, M.R., and Naash, M.I. (2018). Oligomerization of Prph2 and Rom1 is essential for photoreceptor outer segment formation. Hum. Mol. Genet. *27*, 3507–3518. https://doi.org/10.1093/hmg/ddy240.

44. Zhong, X., Gutierrez, C., Xue, T., Hampton, C., Vergara, M.N., Cao, L.H., Peters, A., Park, T.S., Zambidis, E.T., Meyer, J.S., et al. (2014). Generation of three-dimensional retinal tissue with functional photoreceptors from human iPSCs. Nat. Commun. *5*, 4047. https://doi.org/10.1038/ncomms5047.

45. Quinn, P.M., Buck, T.M., Mulder, A.A., Ohonin, C., Alves, C.H., Vos, R.M., Bialecka, M., van Herwaarden, T., van Dijk, E.H.C., Talib, M., et al. (2019). Human iPSC-Derived Retinas Recapitulate the Fetal CRB1 CRB2 Complex Formation and Demonstrate that Photoreceptors and Müller Glia Are Targets of AAV5. Stem Cell Rep. *12*, 906–919. https://doi.org/10.1016/j.stemcr.2019.03.002.

46. Guo, Y., Wang, P., Ma, J.H., Cui, Z., Yu, Q., Liu, S., Xue, Y., Zhu, D., Cao, J., Li, Z., et al. (2019). Modeling Retinitis Pigmentosa: Retinal Organoids Generated From the iPSCs of a Patient With the USH2A Mutation Show Early Developmental Abnormalities. Front. Cell. Neurosci. *13*, 361. https://doi.org/10.3389/fncel.2019.00361.

47. Gao, M.L., Lei, X.L., Han, F., He, K.W., Jin, S.Q., Zhang, Y.Y., and Jin, Z.B. (2020). Patient-Specific Retinal Organoids Recapitulate Disease Features of Late-Onset Retinitis Pigmentosa. Front. Cell Dev. Biol. *8*, 128. https://doi.org/10.3389/fcell.2020.00128.

48. Lane, A., Jovanovic, K., Shortall, C., Ottaviani, D., Panes, A.B., Schwarz, N., Guarascio, R., Hayes, M.J., Palfi, A., Chadderton, N., et al. (2020). Modeling and Rescue of RP2 Retinitis Pigmentosa Using iPSC-Derived Retinal Organoids. Stem Cell Rep. *15*, 67–79. https://doi.org/10.1016/j.stemcr.2020.05.007.

49. Buck, T.M., Quinn, P.M.J., Pellissier, L.P., Mulder, A.A., Jongejan, A., Lu, X., Boon, N., Koot, D., Almushattat, H., Arendzen, C.H., et al. (2023). CRB1 is required for recycling by RAB11A+ vesicles in human retinal organoids. Stem Cell Rep. *18*, 1793–1810. https://doi.org/10.1016/j.stemcr.2023.07.001.

50. Bonnycastle, L.L., Swift, A.J., Mansell, E.C., Lee, A., Winnicki, E., Li, E.S., Robertson, C.C., Parsons, V.A., Huynh, T., Krilow, C., et al. (2024). Generation of Human Isogenic Induced Pluripotent Stem Cell Lines with CRISPR Prime Editing. CRISPR J. *7*, 53–67. https://doi.org/10.1089/crispr.2023.0066.

51. Li, H., Busquets, O., Verma, Y., Syed, K.M., Kutnowski, N., Pangilinan, G.R., Gilbert, L.A., Bateup, H.S., Rio, D.C., Hockemeyer, D., and Soldner, F. (2022). Highly efficient generation of isogenic pluripotent stem cell models using prime editing. eLife *11*, e79208. https://doi.org/10.7554/eLife.79208.

52. Duda, K., Lonowski, L.A., Kofoed-Nielsen, M., Ibarra, A., Delay, C.M., Kang, Q., Yang, Z., Pruett-Miller, S.M., Bennett, E.P., Wandall, H.H., et al. (2014). High-efficiency genome editing via 2A-coupled co-expression of fluorescent proteins and zinc finger nucleases or CRISPR/Cas9 nickase pairs. Nucleic Acids Res. *42*, e84. https://doi.org/10.1093/nar/gku251.

53. Farrar, G.J., Kenna, P., Jordan, S.A., Kumar-Singh, R., Humphries, M.M., Sharp, E.M., Sheils, D.M., and Humphries, P. (1991). A three-base-pair deletion in the peripherin-RDS gene in one form of retinitis pigmentosa. Nature *354*, 478–480. https://doi.org/10.1038/354478a0.

54. Wells, J., Wroblewski, J., Keen, J., Inglehearn, C., Jubb, C., Eckstein, A., Jay, M., Arden, G., Bhattacharya, S., and Fitzke, F. (1993). Mutations in the human retinal degeneration slow (RDS) gene can cause either retinitis pigmentosa or macular dystrophy. Nat. Genet. *3*, 213–218. https://doi.org/10.1038/ng0393-213.

55. Capowski, E.E., Samimi, K., Mayerl, S.J., Phillips, M.J., Pinilla, I., Howden, S.E., Saha, J., Jansen, A.D., Edwards, K.L., Jager, L.D., et al. (2019). Reproducibility and staging of 3D human retinal organoids across multiple pluripotent stem cell lines. Development *146*, dev171686. https://doi.org/10.1242/dev.171686.

56. Chan, C.S.Y., Lonfat, N., Zhao, R., Davis, A.E., Li, L., Wu, M.R., Lin, C.H., Ji, Z., Cepko, C.L., and Wang, S. (2020). Cell type- And stage-specific expression of Otx2 is regulated by multiple transcription factors and cis-regulatory modules in the retina. Development *147*, dev187922. https://doi.org/10.1242/dev.187922.

57. Arikawa, K., Molday, L.L., Molday, R.S., and Williams, D.S. (1992). Localization of peripherin/rds in the disk membranes of cone and rod photoreceptors: relationship to disk membrane morphogenesis and retinal degeneration. J. Cell Biol. *116*, 659–667. https://doi.org/10.1083/jcb.116.3.659.

58. Lindeboom, R.G.H., Supek, F., and Lehner, B. (2016). The rules and impact of nonsense-mediated mRNA decay in human cancers. Nat. Genet. *48*, 1112–1118. https://doi.org/10.1038/ng.3664.

59. Kim, Y.G., Kang, H., Lee, B., Jang, H.J., Park, J.H., Ha, C., Park, H., and Kim, J.W. (2024). A spectrum of nonsense-mediated mRNA decay efficiency along the degree of mutational constraint. Commun. Biol. *7*, 1461. https://doi.org/10.1038/s42003-024-07136-y.

60. Le Hir, H., Gatfield, D., Izaurralde, E., and Moore, M.J. (2001). The exon-exon junction complex provides a binding platform for factors involved in mRNA export and nonsense-mediated mRNA decay. EMBO J. *20*, 4987–4997. https://doi.org/10.1093/emboj/20.17.4987.

61. Tsai, Y.-T., da Costa, B.L., Caruso, S.M., Nolan, N.D., Levi, S.R., Tsang, S.H., and Quinn, P.M.J. (2023). Generation of an Avian Myeloblastosis Virus (AMV) Reverse Transcriptase Prime Editor. Adv. Exp. Med. Biol. *1415*, 109–114. https://doi.org/10.1007/978-3-031-27681-1_17.

62. Patel, A., Garcia Diaz, A., Moore, J.C., Sirabella, D., and Corneo, B. (2020). Establishment and characterization of two iPSC lines derived from healthy controls. Stem Cell Res. *47*, 101926. https://doi.org/10.1016/j.scr.2020.101926.

63. Tso, A., da Costa, B.L., Fehnel, A., Levi, S.R., Jenny, L.A., Ragi, S.D., Li, Y., and Quinn, P.M.J. (2023). Generation of Human iPSC-Derived Retinal Organoids for Assessment of AAV-Mediated Gene Delivery. Methods Mol. Biol. *2560*, 287–302. https://doi.org/10.1007/978-1-0716-2651-1_27.

64. Hebsgaard, S.M., Korning, P.G., Tolstrup, N., Engelbrecht, J., Rouzé, P., and Brunak, S. (1996). Splice site prediction in Arabidopsis thaliana pre-mRNA by combining local and global sequence information. Nucleic Acids Res. *24*, 3439–3452. https://doi.org/10.1093/nar/24.17.3439.

65. Bae, S., Park, J., and Kim, J.S. (2014). Cas-OFFinder: A fast and versatile algorithm that searches for potential off-target sites of Cas9 RNA-guided endonucleases. Bioinformatics *30*, 1473–1475. https://doi.org/10.1093/bioinformatics/btu048.

66. Livak, K.J., and Schmittgen, T.D. (2001). Analysis of relative gene expression data using real-time quantitative PCR and the 2-ΔΔCT method. Methods *25*, 402–408. https://doi.org/10.1006/meth.2001.1262.

OMTN, Volume *36*

Supplemental information

Prime editing for the investigation of aberrant splicing defect associated with a pathogenic *PRPH2* variant

Bruna Lopes da Costa, Kyle M. Helms, Keith Theodore, Yi-Ting Tsai, Salvatore Marco Caruso, Siyuan Liu, Jose Ronaldo Lima de Carvalho, Nicholas D. Nolan, Saleha Tahir, Christopher D. Makinson, Stephen H. Tsang, and Peter M.J. Quinn

Supplemental Material

Supplemental Tables

Table S1 – Spacer and 3'-extension sequences of the pegRNA and nsgRNA constructs

Plasmid name	Spacer (5'-3')	3' Extension (5'-3')
pU6-pegRNA-FANCF+1A>G	GGAATCCCTTCTGCAGCACC	GGAAAAGCGATCCAGGCGCTGCAGAAGGGA
pU6-pegRNA-FANCF+1A>T	GGAATCCCTTCTGCAGCACC	GGAAAAGCGATCCAGGAGCTGCAGAAGGGA
pU6-pegRNA-FANCF+1A>C	GGAATCCCTTCTGCAGCACC	GGAAAAGCGATCCAGGGGCTGCAGAAGGGA
pU6-pegRNA-FANCF+1insA	GGAATCCCTTCTGCAGCACC	GGAAAAGCGATCCAGGTTGCTGCAGAAGG
pU6-pegRNA-FANCF+1insG	GGAATCCCTTCTGCAGCACC	GGAAAAGCGATCCAGGCTGCTGCAGAAGG
pU6-pegRNA-FANCF+1insT	GGAATCCCTTCTGCAGCACC	GGAAAAGCGATCCAGGATGCTGCAGAAGG
pU6-pegRNA-FANCF+1insC	GGAATCCCTTCTGCAGCACC	GGAAAAGCGATCCAGGGTGCTGCAGAAGG
pU6-pegRNA-FANCF+1delA	GGAATCCCTTCTGCAGCACC	GGAAAAGCGATCCAGGGCTGCAGAAGGGA
pU6-pegRNA-FANCF+2delC	GGAATCCCTTCTGCAGCACC	GGAAAAGCGATCCAGTGCTGCAGAAGGGA
pU6-pegRNA-FANCF+2-3delCC	GGAATCCCTTCTGCAGCACC	GGAAAAGCGATCCATGCTGCAGAAGGGA
pU6-pegRNA-FANCF+4delT	GGAATCCCTTCTGCAGCACC	GGAAAAGCGATCCGGTGCTGCAGAAGGGA
pU6-pegRNA-FANCF+5delG	GGAATCCCTTCTGCAGCACC	GGAAAAGCGATCAGGTGCTGCAGAAGGGA
p7SK-nsgRNA-FANCF	GGGGTCCCAGGTGCTGACGT	NOT APPLICABLE
pU6-pegRNA-HEK3+1T>A	GGCCCAGACTGAGCACGTGA	TCCTCTGCCATCTCGTGCTCAGTCTG
pU6-pegRNA-HEK3+1T>C	GGCCCAGACTGAGCACGTGA	TCCTCTGCCATCGCGTGCTCAGTCTG
pU6-pegRNA-HEK3+1T>G	GGCCCAGACTGAGCACGTGA	TCCTCTGCCATCCCGTGCTCAGTCTG
pU6-pegRNA-HEK3+1insA	GGCCCAGACTGAGCACGTGA	TCCTCTGCCATCATCGTGCTCAGTCTG
pU6-pegRNA-HEK3+1insG	GGCCCAGACTGAGCACGTGA	TCCTCTGCCATCACCGTGCTCAGTCTG
pU6-pegRNA-HEK3+1insT	GGCCCAGACTGAGCACGTGA	TCCTCTGCCATCAACGTGCTCAGTCTG
pU6-pegRNA-HEK3+1insC	GGCCCAGACTGAGCACGTGA	TCCTCTGCCATCAGCGTGCTCAGTCTG
pU6-pegRNA-HEK3+1delT	GGCCCAGACTGAGCACGTGA	TCCTCTGCCATCCGTGCTCAGTCTG
pU6-pegRNA-HEK3+2delG	GGCCCAGACTGAGCACGTGA	TCCTCTGCCATACGTGCTCAGTCTG
pU6-pegRNA-HEK3+3delA	GGCCCAGACTGAGCACGTGA	TCCTCTGCCACACGTGCTCAGTCTG
pU6-pegRNA-HEK3+4delT	GGCCCAGACTGAGCACGTGA	TCCTCTGCCTCACGTGCTCAGTCTG
pU6-pegRNA-HEK3+5delG	GGCCCAGACTGAGCACGTGA	TCCTCTGCATCACGTGCTCAGTCTG
pU6-pegRNA-HEK3+1CTTins	GGCCCAGACTGAGCACGTGA	TCCTCTGCCATCAAAGCGTGCTCAGTCTG
p7SK-nsgRNA-HEK3	GTCAACCAGTATCCCGGTGC	NOT APPLICABLE
pU6-pegRNA-PRPH2-828+1G>A-knock-in	GCTCCTCATTTGGCTCTTCG	GGGCCTATCTCGAAGAGCCAAATGAGG

p7SK-nsgRNA-PRPH2-828+1G>A-knock-in	GCTGCTGTAGTAGCTCAGCA	NOT APPLICABLE
pU6-pegRNA1-PRPH2-828+1G>A-correction	GCTCATTTGGCTCTTCGAGAT	GGCCCAGGGCCTACCTCGAAGAGCC
pU6-pegRNA2-PRPH2-828+1G>A-correction	GCTCATTTGGCTCTTCGAGAT	GGCCCAGGGCCTACCTCGAAGAGCCAA
pU6-pegRNA3-PRPH2-828+1G>A-correction	GCTCATTTGGCTCTTCGAGAT	GGCCCAGGGCCTACCTCGAAGAGCCAAATG
pU6-pegRNA4-PRPH2-828+1G>A-correction	GCTCATTTGGCTCTTCGAGAT	CCCAGCTGGCCCAGGGCCTACCTCGAAGAGCC
pU6-pegRNA5-PRPH2-828+1G>A-correction	GCTCATTTGGCTCTTCGAGAT	CCCAGCTGGCCCAGGGCCTACCTCGAAGAGCCAA
pU6-pegRNA6-PRPH2-828+1G>A-correction	GCTCATTTGGCTCTTCGAGAT	CCCAGCTGGCCCAGGGCCTACCTCGAAGAGCCAAATG
pU6-nsgRNA1-PRPH2-828+1G>A-correction	GCCTTACCCTCTACCCCCAGC	NOT APPLICABLE
pU6-nsgRNA2-PRPH2-828+1G>A-correction	GAGGAGCGTGACGACACCCA	NOT APPLICABLE
pU6-nsgRNA3-PRPH2-828+1G>A-correction	GTTAGACCCAAATGGGACCGG	NOT APPLICABLE
pU6-nsgRNA4-PRPH2-828+1G>A-correction	GACAGGTTGAGCTCCTCCGTC	NOT APPLICABLE

Table S2 – Primer sequences employed for amplifying pegRNA and nsgRNA in the cloning of all-in-one constructs

All-in-one Type	Forward primer (5'-3')	Reverse primer (5'-3')
T1 pU6-pegRNA-HEK3+1CTTins	GCGCTGCTTCGATTTACCTGGAGATGACACGGGT	CTGGCCCGTACATCGTTCG
T1 p7SK-nsgRNA-HEK3	CGATGTACGGGCCAGTTTTGCGCTGCTTCGAGCG	CCCGGGAAGTTATTTGTGTCATCTCCAGGTCCACG
T2 pU6-pegRNA-HEK3+1CTTins	GCGCTGCTTCGATTTACCTGGAGATGACACGGGT	CTGGCCCGTACATCGTTCG
T2 p7SK-nsgRNA-HEK3	CCCGGGAAGTTATTTTGCGCTGCTTCGAGCG	CGATGTACGGGCCAGGTGTCATCTCCAGGTCCACG
T3 pU6-pegRNA-HEK3+1CTTins	ACCTGGAGATGACACGGGT	GCGCTGCTTCGATTTCTGGCCCGTACATCGTTCG
T3 p7SK-nsgRNA-HEK3	CCCGGGAAGTTATTTTGCGCTGCTTCGAGCG	GTGTCATCTCCAGGTGTCATCTCCAGGTCCACG

T4 pU6-pegRNA-HEK3+1CTTins	ACCTGGAGATGACACGGGT	GCGCTGCTTCGATTTCTGGCCCGTAC ATCGTTCG
T4 p7SK-nsgRNA-HEK3	GTGTCATCTCCAGGTTTTGCGCTGCTT CGAGCG	CCCGGGAAGTTATTTGTGTCATCTCC AGGTCCACG
T5 pU6-pegRNA-HEK3+1CTTins	GCAGGAAAGAACATGACCTGGAGAT GACACGGGT	CTGTCGCCGCACATGCTGGCCCGTAC ATCGTTCG
T6 p7SK-nsgRNA-HEK3	GCAGGAAAGAACATGTTTTGCGCTGC TTCGAGCG	CTGTCGCCGCACATGTGTCATCTCCA GGTCCACG
pU6-pegRNA-HEK3	GCGCTGCTTCGATTTACCTGGAGATG ACACGGGT	CTGGCCCGTACATCGTTCG
p7SK-nsgRNA-HEK3	CGATGTACGGGCCAGTTTTGCGCTGC TTCGAGCG	CCCGGGAAGTTATTTGTGTCATCTCC AGGTCCACG
pU6-pegRNA-FANCF	GCGCTGCTTCGATTTACCTGGAGATG ACACGGGT	CTGGCCCGTACATCGTTCG
p7SK-nsgRNA-FANCF	CGATGTACGGGCCAGTTTTGCGCTGC TTCGAGCG	CCCGGGAAGTTATTTGTGTCATCTCC AGGTCCACG
pU6-pegRNA-PRPH2-828+1G>A-knock-in	GCGCTGCTTCGATTTACCTGGAGATG ACACGGGT	CTGGCCCGTACATCGTTCG
p7SK-nsgRNA-PRPH2-828+1G>A-knock-in	CGATGTACGGGCCAGTTTTGCGCTGC TTCGAGCG	CCCGGGAAGTTATTTGTGTCATCTCC AGGTCCACG
pU6-pegRNA2-PRPH2-828+1G>A-correction	GCGCTGCTTCGATTTGATCCG GTACCAAGGTCGGG	CTGGCCCGTACATCGTACCTC GAGCGGCCCAA
pU6-nsgRNA3-PRPH2-828+1G>A-correction	CGATGTACGGGCCAGACCAAG GTCGGGCAGGAA	CCCGGGAAGTTATTCGAGCG GCCCAAGCTTA

Table S3 – Primers used for genomic amplification

Target	Forward primer (5'-3')	Reverse primer (5'-3')
PRPH2	CACCAACAACTCAGCACACTAC	TCCACTGAAGGCTGTTTCCAA
PRPH2-NGS-Barcode1	ACACTCTTTCCCTACACGACGCTCTTCCG ATCTGGAACACCAGACGGAGGAGCTCA	GACTGGAGTTCAGACGTGTGCTCTTCCGATCTCA GAGAGGGAGGCATGCTCTCCA
PRPH2-NGS-Barcode2	ACACTCTTTCCCTACACGACGCTCTTCCG ATCTACCACACCAGACGGAGGAGCTCA	GACTGGAGTTCAGACGTGTGCTCTTCCGATCTTCA GGAGGGAGGCATGCTCTCCA
PRPH2-NGS-Barcode3	ACACTCTTTCCCTACACGACGCTCTTCCG ATCTATGGCACCAGACGGAGGAGCTCA	GACTGGAGTTCAGACGTGTGCTCTTCCGATCTGA TGGAGGGAGGCATGCTCTCCA
PRPH2-NGS-Barcode4	ACACTCTTTCCCTACACGACGCTCTTCCG ATCTCTACCACCAGACGGAGGAGCTCA	GACTGGAGTTCAGACGTGTGCTCTTCCGATCTTAC CGAGGGAGGCATGCTCTCCA
PRPH2-NGS-Barcode5	ACACTCTTTCCCTACACGACGCTCTTCCG ATCTAGTCCACCAGACGGAGGAGCTCA	GACTGGAGTTCAGACGTGTGCTCTTCCGATCTTG GTGAGGGAGGCATGCTCTCCA
PRPH2-NGS-Barcode6	ACACTCTTTCCCTACACGACGCTCTTCCG ATCTGTCTCACCAGACGGAGGAGCTCA	GACTGGAGTTCAGACGTGTGCTCTTCCGATCTCCT TGAGGGAGGCATGCTCTCCA
FANCF	CAGTACGCAGAGAGTCGCC	CGACCAAAGCGCCGATGG
FANCF-NGS-Barcode1	ACACTCTTTCCCTACACGACGCTCTTCCG ATCTGGAAGAACAGTACGCAGAGAGTC GCC	GACTGGAGTTCAGACGTGTGCTCTTCCGATCTGA CGGAACGACCAAAGCGCCGATGG

FANCF-NGS-Barcode2	ACACTCTTTCCCTACACGACGCTCTTCCGATCTCAGAGAACAGTACGCAGAGAGTCGCC	GACTGGAGTTCAGACGTGTGCTCTTCCGATCTAGTGGAACGACCAAAGCGCCGATGG
FANCF-NGS-Barcode3	ACACTCTTTCCCTACACGACGCTCTTCCGATCTACCAGAACAGTACGCAGAGAGTCGCC	GACTGGAGTTCAGACGTGTGCTCTTCCGATCTTGGCGAACGACCAAAGCGCCGATGG
FANCF-NGS-Barcode4	ACACTCTTTCCCTACACGACGCTCTTCCGATCTCCAGGAACAGTACGCAGAGAGTCGCC	GACTGGAGTTCAGACGTGTGCTCTTCCGATCTCTCCGAACGACCAAAGCGCCGATGG
HEK3	ATGTGGGCTGCCTAGAAAGG	GCCCAGCCAAACTTGTCAACC
HEK3-NGS-Barcode1	ACACTCTTTCCCTACACGACGCTCTTCCGATCTGGAAGAAATGTGGGCTGCCTAGAAAGG	GACTGGAGTTCAGACGTGTGCTCTTCCGATCTGACGGAAGCCCAGCCAAACTTGTCAACC
HEK3-NGS-Barcode2	ACACTCTTTCCCTACACGACGCTCTTCCGATCTCAGAGAAATGTGGGCTGCCTAGAAAGG	GACTGGAGTTCAGACGTGTGCTCTTCCGATCTAGTGGAAGCCCAGCCAAACTTGTCAACC
HEK3-NGS-Barcode3	ACACTCTTTCCCTACACGACGCTCTTCCGATCTACCAGAAATGTGGGCTGCCTAGAAAGG	GACTGGAGTTCAGACGTGTGCTCTTCCGATCTTGGCGAAGCCCAGCCAAACTTGTCAACC
HEK3-NGS-Barcode4	ACACTCTTTCCCTACACGACGCTCTTCCGATCTCCAGGAAATGTGGGCTGCCTAGAAAGG	GACTGGAGTTCAGACGTGTGCTCTTCCGATCTCTCCGAAGCCCAGCCAAACTTGTCAACC
KI-pegRNA-OT1-CHRNB4	ACGAGGTGGTGCTTTCCCT	CACTTGGCAAGGGATCGGG
KI-pegRNA-OT2-No coding	AGCAGTAATGCTAGGGGCT	TAGGGCTTCTAGGCCTGGC
KI-pegRNA-OT3-NCAM2	TGGTGCAGCCAGAAAAAGAATGC	TTTGGCCAAGGTAAGGACATTGG
KI-pegRNA-OT4-ZFHX3	TGGGACTGGAAGGTCACCC	CACATGCGATGGAGGGTGC
KI-pegRNA-OT5-IFT43	TGTGGGAACTTGGTTTCGGAC	CATTGCAAGGGAGGCAGAGT
KI-pegRNA-OT6-No coding	CCACAATTACTTCTGCACCAACC	CCTGCTGCACTGAGTTACCA
KI-nsgRNA-OT1-DMTN	AACTACCACCTCTAGGCAAATGG	AGGTCGTAATCTGTTGGCAACAG
KI-nsgRNA-OT2-No coding	GAGGAACAGGGGCATGCAC	ACCCAGCCACTTGTTCCCT
KI-nsgRNA-OT3-ZNF286A/TBC1D26	GCCTTACACAGCCATGCCA	AGCCGTCTGACTTGAGGTG
KI-nsgRNA-OT4-TBC1D27P	GGGAGGCAGGAGGACACTC	ACAGTCATGCCAGGGGACA
KI-nsgRNA-OT5-RGS3	GCTGACAGGACACAGCCTG	CGCAGCAAAAGCCAGGAGA
KI-nsgRNA-OT6-UBA1	ACAGGAAGGCGTGCTCACT	AGGCCCAGTGGGTTGGAAT
KI-nsgRNA-OT7-No coding	GGGAAGGTGCTCAGAAGCC	CTTGGGCTCCCCTTTGCTG
KI-nsgRNA-OT8-SWAP70	TGATTCAGCAGATCGTTAGACTGG	GTCACCCTAACCAAGCATTTATCA
KI-nsgRNA-OT9-RAB6B	CTGTCCGGTGGGGATGAGT	TGTGAGGTGTGATGGGGTTG

*OT stands for off targeting

Table S4 – Primers and probes used for real-time PCR

Target	Forward primer (5'-3')	Reverse primer (5'-3')	Probes (5'-3')
Total PRPH2 Transcript	GAACTCCATGGGTGTCGTC	CCAGCGACGTCTGTAGGTA	**5HEX/** ACGCTCCTC **/ZEN/** ATTTGGCTCTTCGAG **/3IABkFQ/**
Canonical PRPH2 Transcript	GAACTCCATGGGTGTCGTC	CCAGCGACGTCTGTAGGTA	**5SUN/** TTGGCTCTT **/ZEN/** CGAGGTGACCATTACAA **/3IABkFQ/**
Mutant PRPH2 Transcript	GAACTCCATGGGTGTCGTC	CCAGCGACGTCTGTAGGTA	**56-FAM/** TTGGCTCTT **/ZEN/** CGAGATAGGCCCTGG **/3IABkFQ/**
β-actin	ACAGAGCCTCGCCTTTG	CCTTGCACATGCCGGAG	**56-FAM/** TCATCCATG **/ZEN/** GTGAGCTGGCGG **/3IABkFQ/**

Supplemental Figures

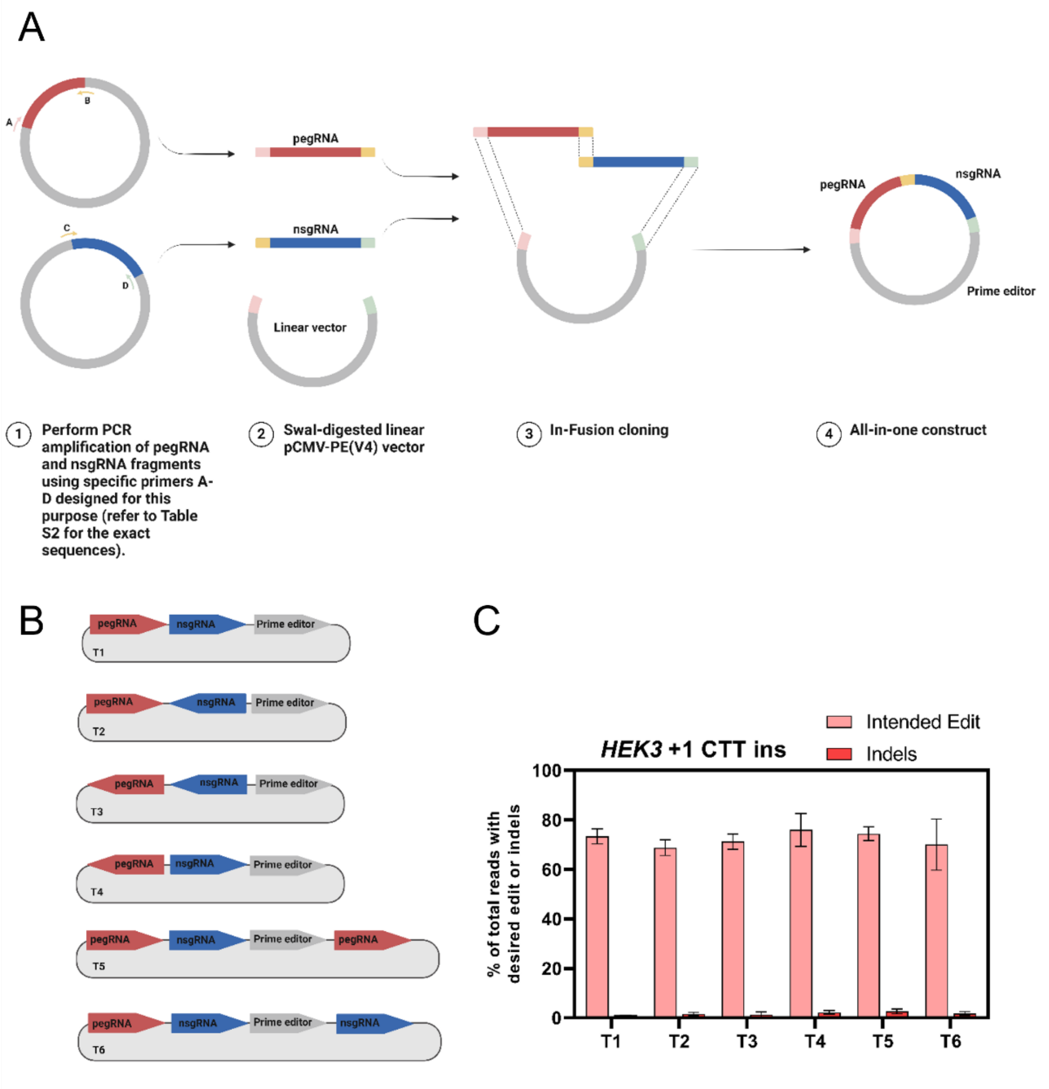

Figure S1. Development of an All-in-One Prime Editing System. (A) Overview of the all-in-one assembly protocol, which includes four steps: Step 1 involves PCR amplification of the pegRNA and nsgRNA fragments. Step 2 consists of linearizing the pCMV-PE(V4) vector using the *SwaI* restriction enzyme. Step 3 is the infusion cloning process that leads to the generation of the all-in-one construct in Step 4. (B) Designs for our all-in-one system. In the T1-T4 designs, we modified the orientation of the pegRNA, nsgRNA, and their respective promoters. In T5 and T6, we enhanced the expression levels of pegRNA and nsgRNA, respectively. (C) PE editing efficiency of T1-T6 all-in-one designs for installing the HEK3 +1CTTins in HEK293T cells. Data are presented as mean ± SD (n=2). Schematics created using Biorender.

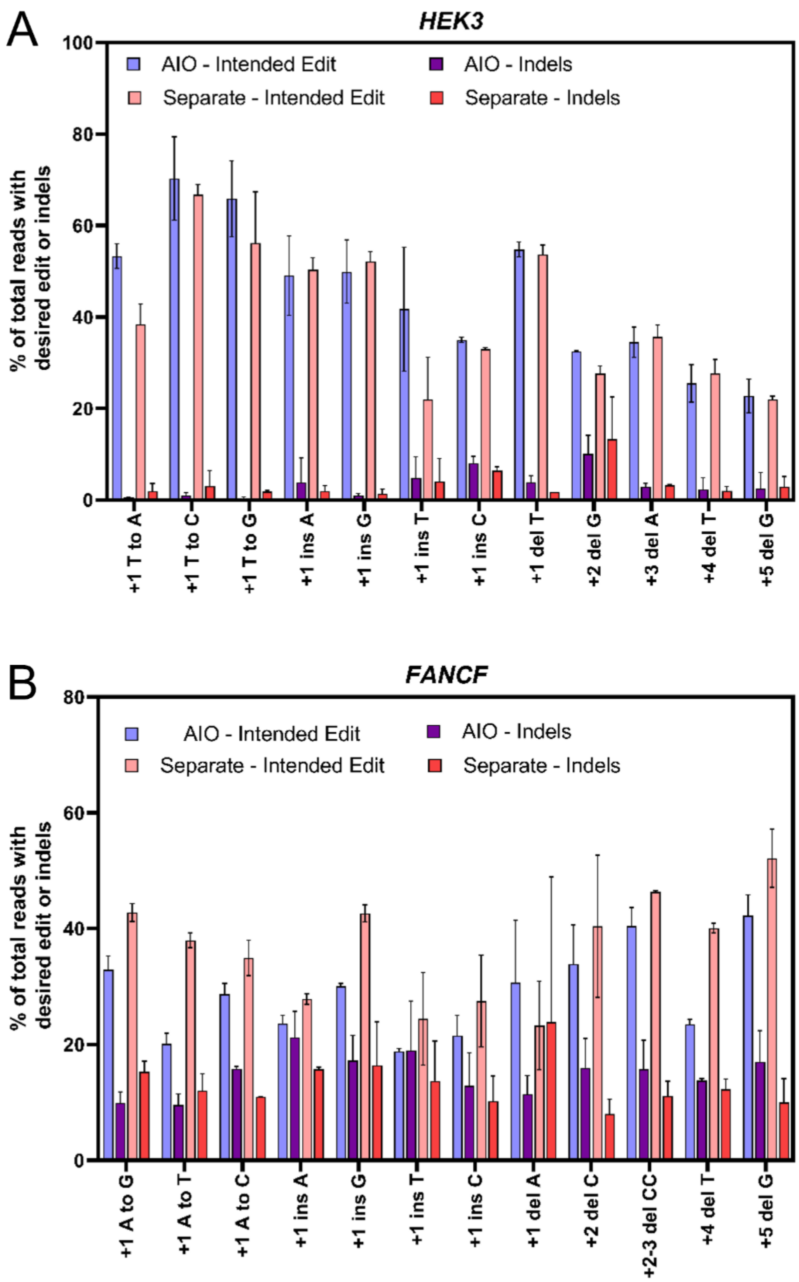

Figure S2. All-in-One PE Demonstrates Comparable Editing Efficiency to Separate Systems. Comparison of separate and all-in-one systems for different types of edits in the (A) *HEK3* and (B) *FANCF* locus. Data are presented as mean ± SD (n=2).

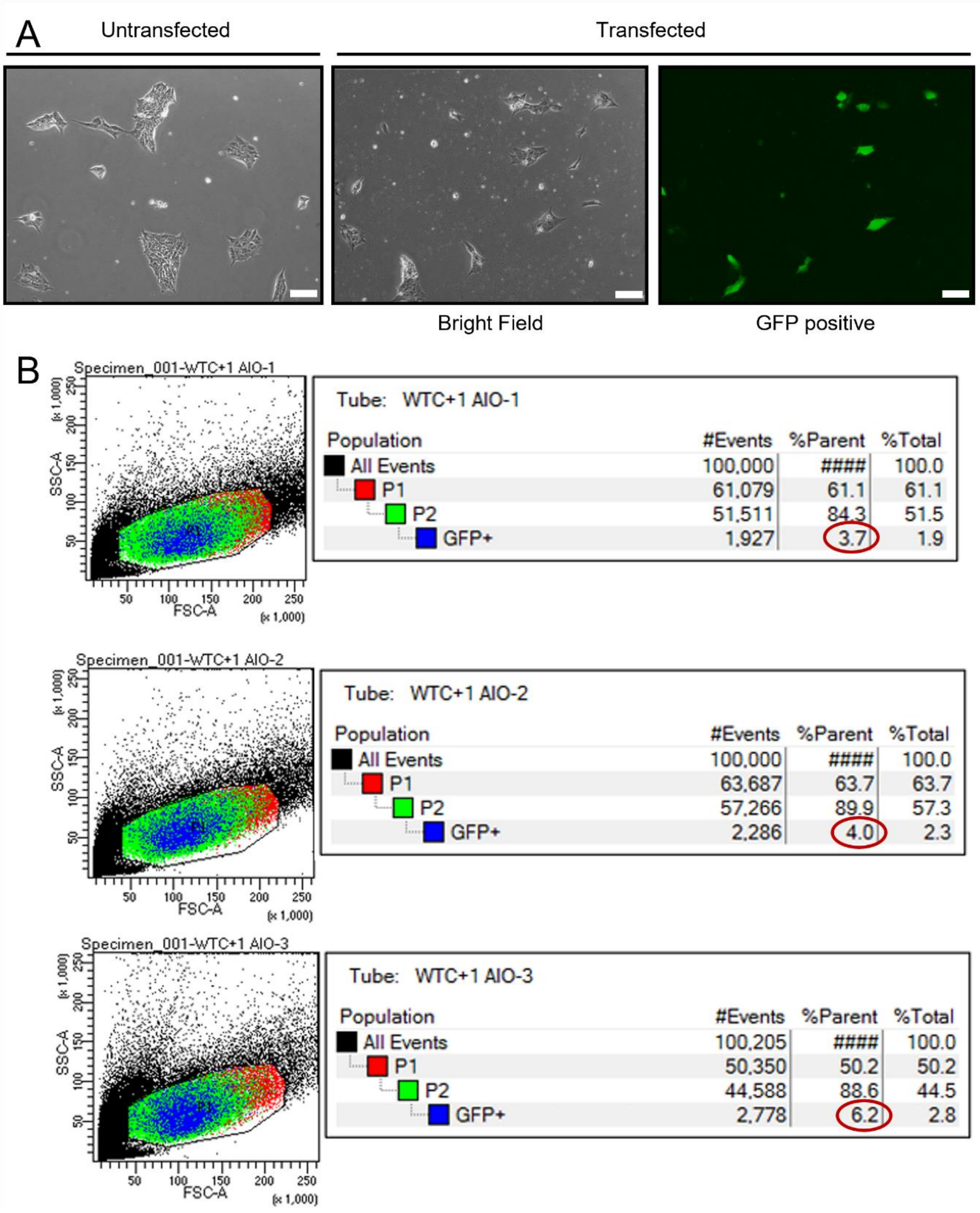

Figure S3. Delivery of the All-in-One PE System for Introducing the *PRPH2* c.828+1G>A Variant in hiPSCs. (A) Images of untransfected hiPSCs and hiPSCs transfected using Lipofectamine™ Stem. Representative images are shown, with a scale bar indicating 100 µm. (B) Quantification of GFP-positive hiPSCs (red circles) sorted by FACS, presented as triplicate measurements.

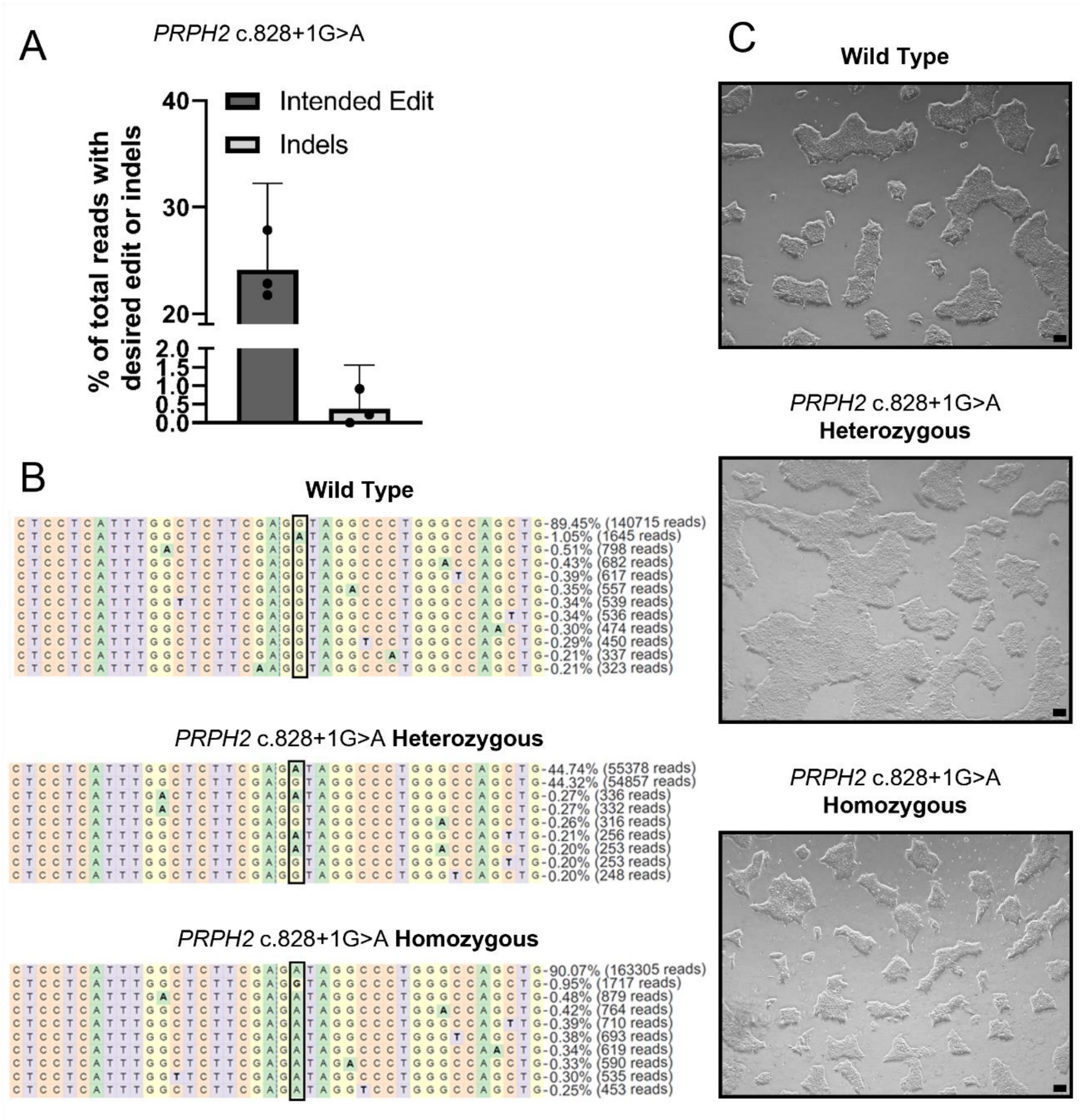

Figure S4. Efficient Installation of the *PRPH2* c.828+1G>A Variant in hiPSC. (A) Quantification of the percentage of total reads containing the desired edits or indels, assessed by next-generation sequencing from pooled GFP-positive cells at 6 days post-transfection. Data are presented as mean ± 95% CI (n=3). (B) Next-generation sequencing validation confirming the successful introduction of the *PRPH2* c.828+1G>A variant in WTC11 hiPSCs, detected in both heterozygous and homozygous states. (C) Bright-field images showing the normal cellular morphology of *PRPH2* c.828+1G>A wild-type, heterozygous, and homozygous hiPSCs. Representative images are shown, with a scale bar indicating 100 µm.

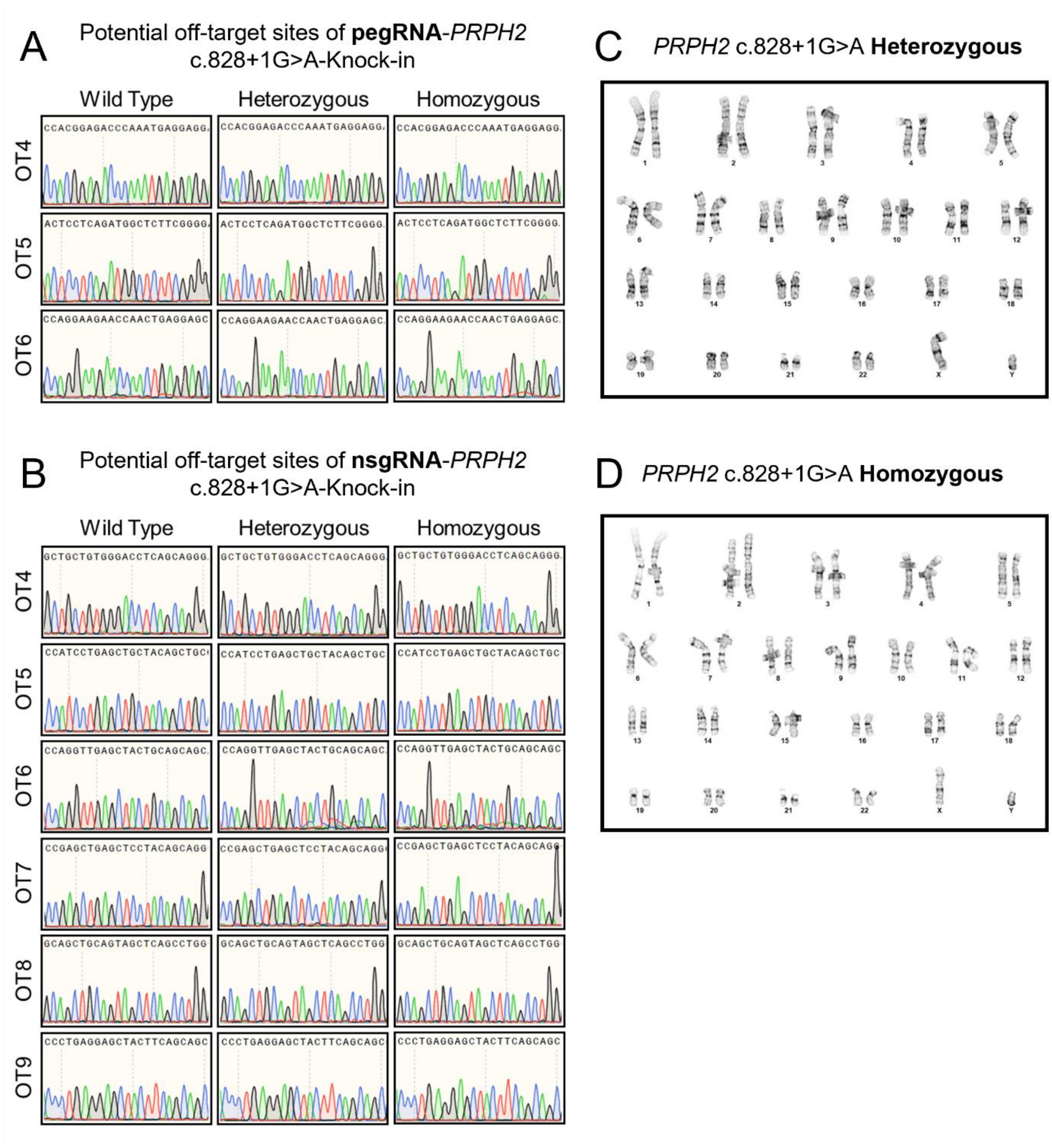

Figure S5. Prime Editing for the *PRPH2* c.828+1G>A Variant in hiPSC Shows no Off-Target Effects or Chromosomal Abnormalities. (A-B) Dideoxy sequencing traces indicate no off-target effects in heterozygous and homozygous clones at (A) Remaining three potential off-target sites associated with pegRNA-*PRPH2* c.828+1G>A knock-in. (B) Remaining six potential off-target sites associated with nsgRNA-*PRPH2* c.828+1G>A knock-in. (C-D) Karyotype analysis shows no chromosomal abnormalities in edited clones: (C) heterozygous and (D) homozygous.

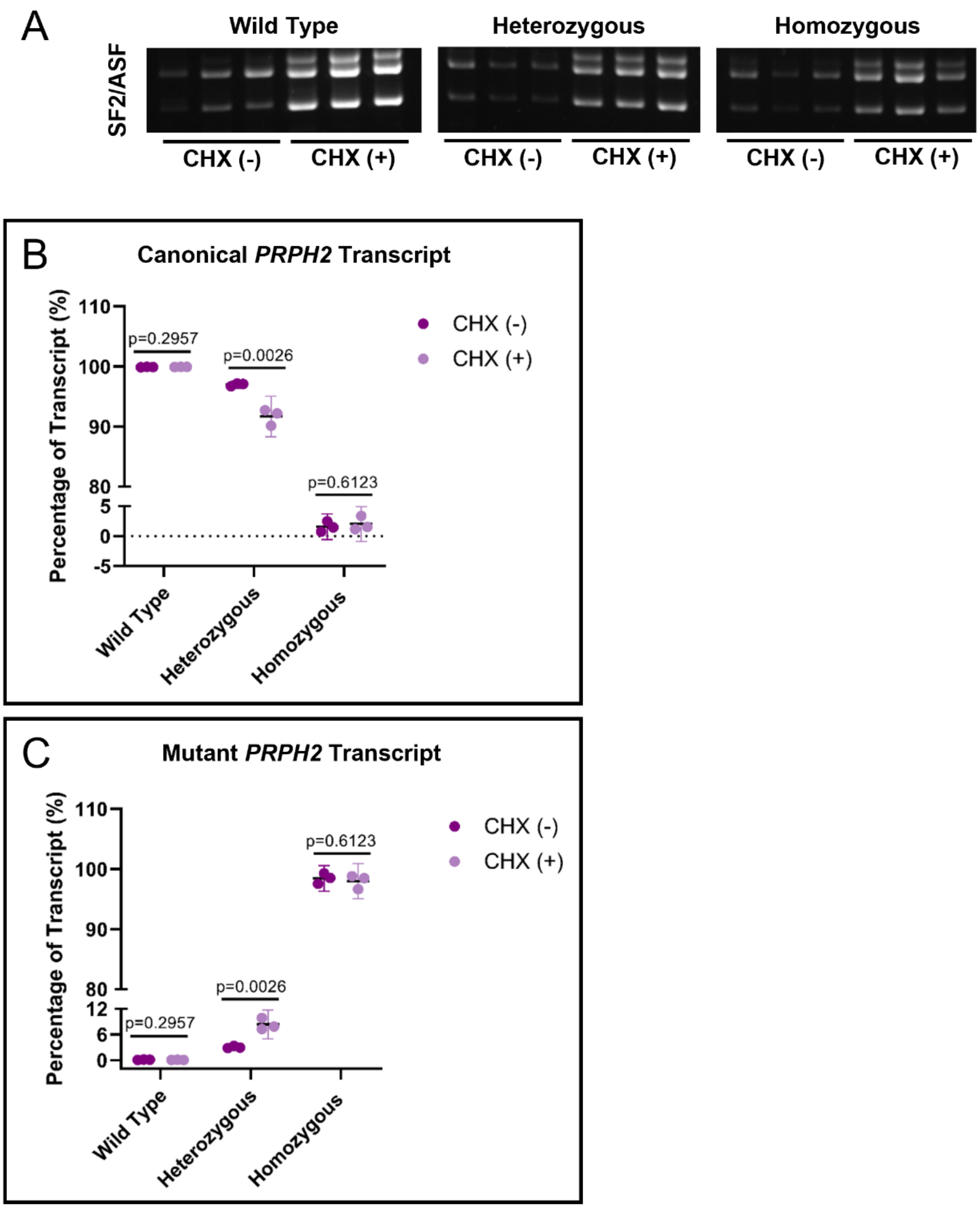

Figure S6. Cycloheximide (CHX) Prevents Nonsense-mediated Decay of the Mutant *PRPH2* Transcript in the *PRPH2* c.828+1G>A Heterozygous hiPSC. (A) Representative agarose gel confirming increased SF2/ASF transcript expression as a positive control for CHX treatment. (B-C) Quantification of *PRPH2* transcript levels by real-time PCR. (B) Canonical *PRPH2* transcript levels, and (C) mutant *PRPH2* transcript levels. Data are presented as mean ± 95% CI (n=3).

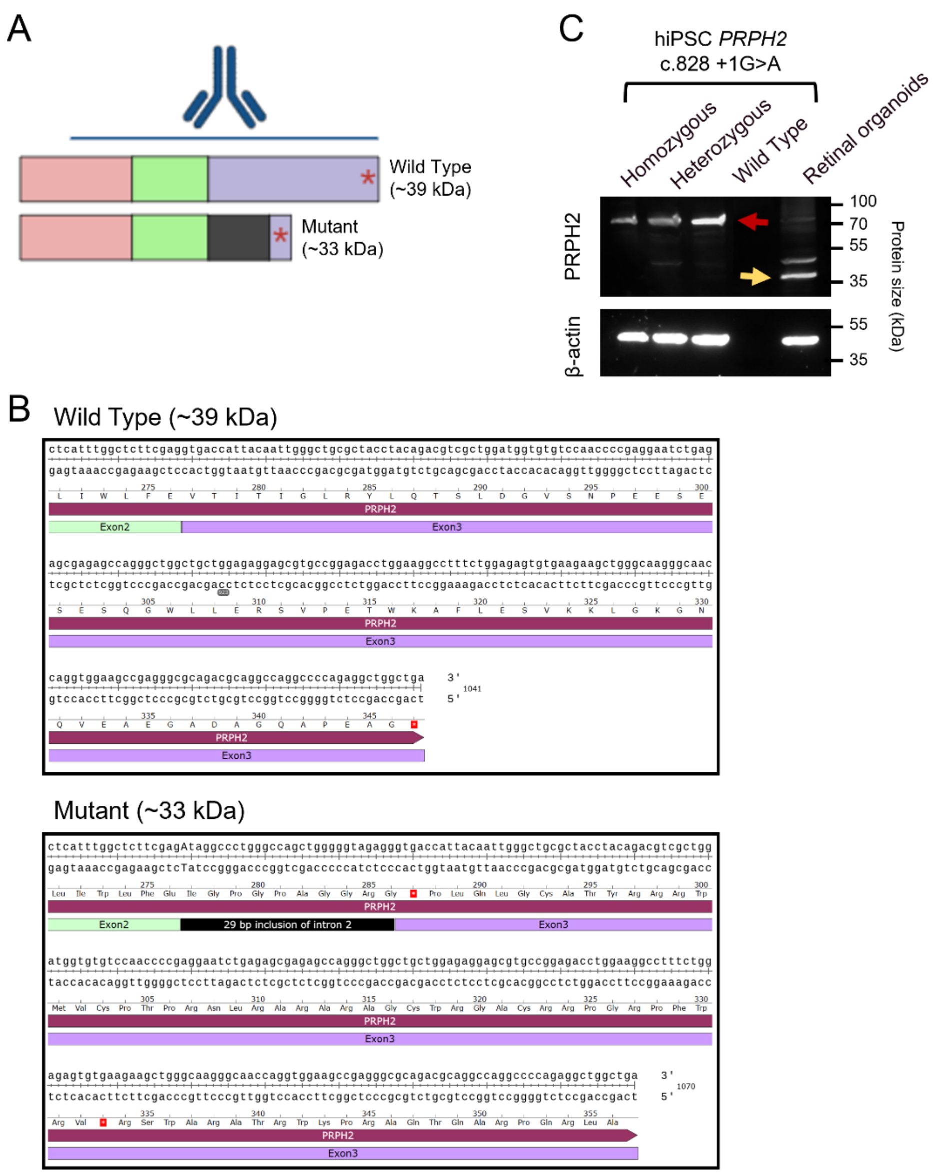

Figure S7. PRPH2 Protein Presence in hiPSCs and Retinal Organoids. (A) Schematic representation of the PRPH2 protein structure highlighting the antibody binding epitope in the wild-type and mutant forms. Red asterisks illustrate the stop-codon position. (B) SnapGene analysis predicts that the *PRPH2* c.828+1G>A mutation results in a truncated protein product. (C) Western blot analysis showing PRPH2 protein presence in hiPSCs and wild type retinal organoids (DD225). In hiPSCs, PRPH2 is detected predominantly as dimeric form (red arrow), whereas in retinal organoids, the monomeric form is more prominent (yellow arrow). Schematic created using Biorender.

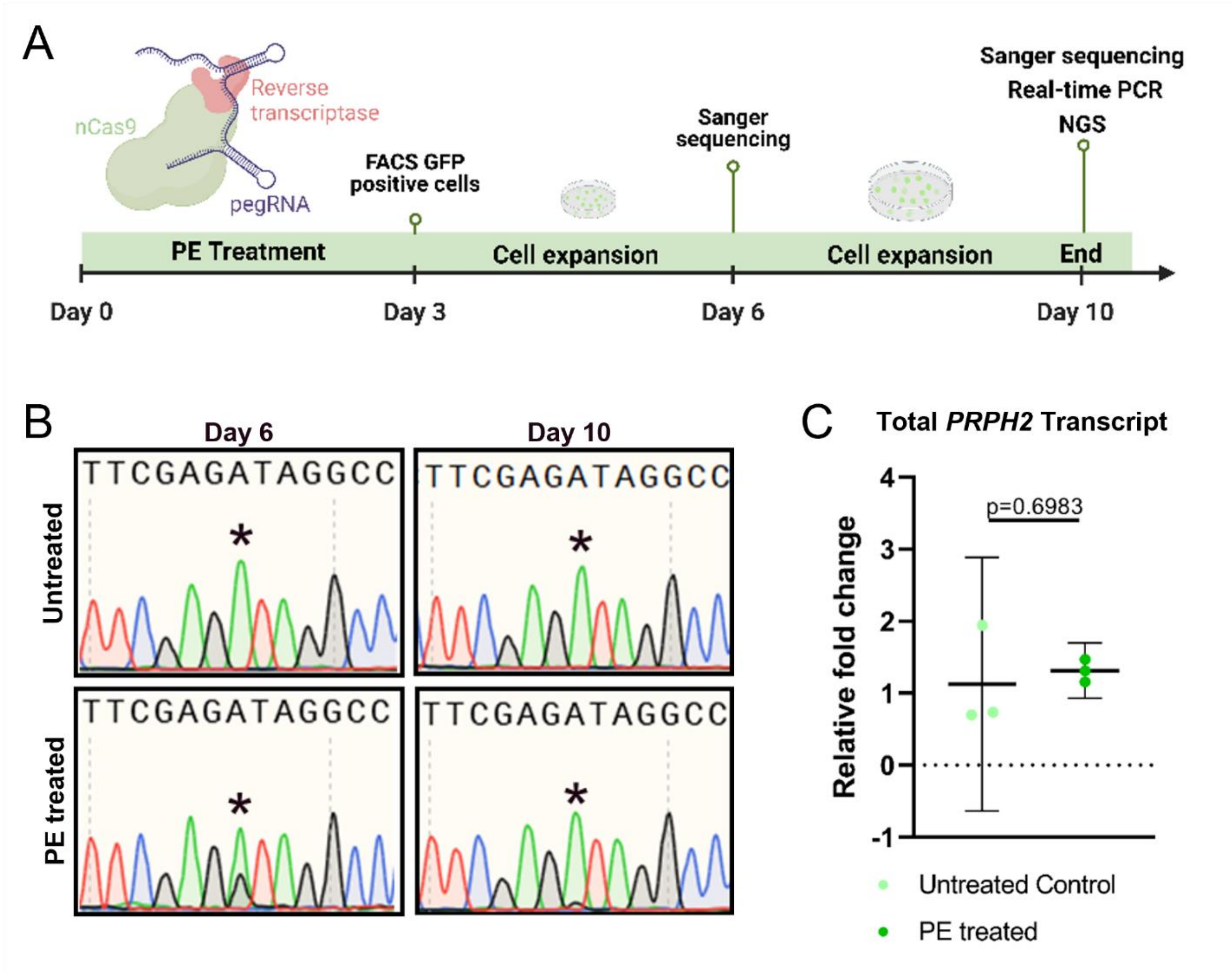

Figure S8. Prime Editing Corrects the *PRPH2* c.828+1G>A Mutation in hiPSCs. (A) Illustration of the experimental design (B) Dideoxy traces demonstrating the correction of homozygous *PRPH2* c.828+1G>A hiPSCs at 6 and 10 days following prime editing treatment. (C) Quantification of total *PRPH2* transcripts by real-time PCR. Data is expressed as mean ± 95% CI (n=3). Untreated Control = c.828+1G>A homozygous line, PE treated = the treated c.828+1G>A homozygous line that shows wild type genotype. Schematic created using Biorender.
